# Sex Hormones and Risk of Aneurysmal Subarachnoid Hemorrhage: A Mendelian Randomization Study

**DOI:** 10.1161/STROKEAHA.121.038035

**Published:** 2022-06-02

**Authors:** Rob Molenberg, Chris H.L. Thio, Marlien W. Aalbers, Maarten Uyttenboogaart, ISGC Intracranial Aneurysm Working Group, Susanna C. Larsson, Mark K. Bakker, Ynte M. Ruigrok, Harold Snieder, J. Marc C. van Dijk

**Affiliations:** Departments of Neurosurgery (R.M., M.W.A., J.M.C.v.D.), University of Groningen, University Medical Center Groningen, the Netherlands.; Epidemiology (C.H.L.T., H.S.), University of Groningen, University Medical Center Groningen, the Netherlands.; Neurology and Medical Imaging Center (M.U.), University of Groningen, University Medical Center Groningen, the Netherlands.; Unit of Cardiovascular and Nutritional Epidemiology, Institute of Environmental Medicine, Karolinska Institutet, Stockholm, Sweden (S.C.L.).; Unit of Medical Epidemiology, Department of Surgical Sciences, Uppsala University, Sweden (S.C.L.).; Department of Neurology and Neurosurgery, UMC Utrecht Brain Center, University Medical Center Utrecht, University Utrecht, the Netherlands (M.K.B., Y.M.R.).

**Keywords:** hormones, menopause, sex hormone-binding globulin, subarachnoid hemorrhage, testosterone

## Abstract

The risk of aneurysmal subarachnoid hemorrhage (aSAH) is increased in postmenopausal women compared with men of similar age, suggesting a role for sex hormones. We aimed to explore whether sex hormones, and age at menarche/menopause have a causal effect on aSAH risk by conducting a 2-sample MR study (Mendelian randomization).

**Methods:**

We obtained sex-specific genetic instruments for serum estradiol, bioavailable testosterone (BioT), SHBG (sex hormone-binding globulin), and age at menarche/menopause from genome-wide association studies. The associated sex-specific aSAH risk was estimated with inverse-variance weighted MR analyses with various statistical sensitivity analyses. Multivariable and cluster MR analyses were performed for BioT and SHBG to account for a genetic and phenotypic correlation between the 2 exposures. The clusters represented (1) single-nucleotide polymorphisms primarily increasing SHBG, with secondary decreasing effects on BioT, and (2) single-nucleotide polymorphisms affecting BioT without affecting SHBG.

**Results:**

Univariable MR analyses showed an 18% increased aSAH risk among women per 1-SD increase in genetically determined SHBG levels (odds ratio, 1.18 [95% CI, 1.05–1.34]; *P*=0.007). Suggestive evidence was identified for a 27% decreased risk of aSAH among women per 1-SD increase in BioT (odds ratio, 0.73 [95% CI, 0.55–0.95]; *P*=0.02). The latter association disappeared in cluster analysis when only using SHBG-independent variants. MR analyses with variants from the cluster with primary SHBG effects and secondary (opposite) BioT-effects yielded a statistically significant association (odds ratio, 1.21 [95% CI, 1.05–1.40]; *P*=0.008). No other causal associations were identified.

**Conclusions:**

Genetic predisposition to elevated serum levels of SHBG, with secondary lower serum BioT levels, is associated with an increased aSAH risk among women, suggesting that SHBG and BioT causally elevate aSAH risk. Further studies are required to elucidate the underlying mechanisms and their potential as an interventional target to lower aSAH incidence.

Aneurysmal subarachnoid hemorrhage (aSAH) can have devastating health effects.^1^ The incidence of aSAH rises with age and remains similar between men and women for the most part of an average lifespan. However, epidemiological data demonstrate that the incidence of aSAH among women disproportionally increases around the start of natural menopause, at the approximate age of 50.^2^ Since exposure to estrogens decreases substantially after menopause it is often suggested that estradiol, among other sex hormones, may influence the risk on aSAH.^3,4^ In this respect, SHBG (sex hormone-binding globulin) may also play a role. SHBG is a protein synthesized and secreted by the liver. Once it reaches the bloodstream, it can bind to androgens and estrogens with high affinity. By doing so, it regulates the bioavailability of these steroids, which is usually defined as the percentage of androgens or estrogens not bound to SHGB but either free or loosely bound to albumin.

Until now, most attention has been focused on the role of estradiol (the most potent estrogen), considering its ability to suppress vascular inflammation as a known driver of intracranial aneurysm progression.^5,6^ However, while experimental studies frequently indicate a substantial role of estradiol on aSAH risk, observational studies on the association between estradiol among other sex hormones and aSAH are still indecisive.

MR studies (Mendelian randomization) use genetic variants (single-nucleotide polymorphisms [SNPs]) associated with an exposure to investigate its effect on an outcome. The random allocation of SNP alleles at conception offers an opportunity to potentially overcome limitations inherent to traditional observational studies, such as residual confounding.^7^ Analogous to randomized controlled trials, the principle of randomization is exploited to ascertain an unconfounded and therefore causal exposure-outcome relationship. Therefore, we aimed to explore the association between sex hormones and the risk of aSAH using an MR approach. In particular, we assessed whether a genetically determined increase in age at menarche (AAM), age at natural menopause, serum bioavailable testosterone (BioT), SHBG, and estradiol influences the sex-specific risk of aSAH.

## Methods

### Data Availability

We used summary data from published studies for our analyses, publicly available via the original studies (data sources in the Supplemental Material). All studies obtained relevant ethical approval and participant consent.

### Exposure Data

We retrieved publicly available sex-specific genome-wide association study (GWAS) summary-level data of SNPs associated with the exposures (Table).^9–11^ We selected SNPs associated with the exposure at the genome-wide significance level (*P*<5×10^−8^). Independence of SNPs was assessed using stringent criteria (r^2^, 0.001; clumping window, 10 000 kb). If an instrumental SNP for the exposure was not available in the outcome data set, we replaced it with a suitable proxy SNP (r^2^>0.8 in the European 1000 Genomes Project reference panel using LDlink [https://ldlink.nci.nih.gov/]) or removed it in the absence of such a proxy. We harmonized the SNP alleles across studies and removed palindromic SNPs with ambiguous allele frequencies (0.42–0.58). Data of all instrumental variables used are shown in Table S1 through S6. For estradiol, we used a single SNP (rs727479) which has been reported in the literature and was found to be associated with serum estradiol levels in both men and postmenopausal women, although with different effect sizes (Table S7).^12,13^ The SNP is located in the CYP19A1 gene, which encodes aromatase, the enzyme responsible for converting testosterone to estradiol.

**Table.**
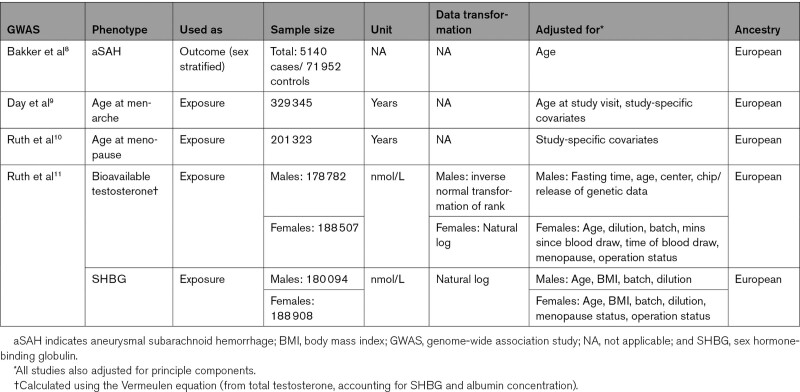
Characteristics of the Used GWAS

### Outcome Data

We obtained sex-specific summary-level outcome data from a GWAS on aSAH, comprising 23 cohorts with a total of 5140 cases and 71 934 controls.^8^ All individuals were of European ancestry. As the calculated potential UK Biobank sample overlap between exposure and outcome GWASs was <4% at maximum, we considered the risk of bias due to sample overlap minimal.^14^

### Statistical Analysis

We performed primary MR analysis among women using a random-effects inverse-variance weighted meta-analysis, if at least 2 SNPs for the exposure were available.^15^ This comprises a meta-analysis of SNP-specific Wald estimates (ie, SNP-outcome β divided by the SNP-exposure β). This method assumes that all variants are valid instruments.^16^ Consequently, we performed several statistical sensitivity analyses. We first tested for heterogeneity between variant-specific estimates using Cochran Q statistic in the inverse-variance weighted model. We then explored horizontal pleiotropy via the MR-Egger and MR-Pleiotropy Residual Sum and Outlier methods.^17,18^ A significant nonzero Egger intercept is suggestive of unbalanced, directional horizontal pleiotropy. It can thereby identify and correct for bias due to directional pleiotropic effects, assuming that the size of the pleiotropic effects is independent of the SNP-exposure effects (InSIDE-assumption). The MR-Egger estimate is however sensitive to outliers and influential data points, which may lead to low statistical power in estimating a causal effect. MR-Pleiotropy Residual Sum and Outlier calculates the effect estimate after identifying and excluding outlier SNPs (ie, potential pleiotropic variants) if present. We additionally performed a weighted median-based MR analysis, which assumes that at least half of the included variants are valid instrumental variables. We repeated the analyses after MR-Steiger filtering which removes SNPs suggestive of a reversed causal direction (ie, those explaining more variance in the outcome than in exposure), a violation of the exclusion restriction assumption.^19^ In addition, we conducted sensitivity MR analyses for all exposures using the male-specific exposure and outcome summary data. In this respect, AAM and age at natural menopause functioned as negative control outcomes. For estradiol with only one SNP available, we calculated the Wald estimate, as other MR methods require at least 2 SNPs.

A strong genetic correlation (r_g_=−0.74) was reported between SHBG and BioT among women.^11^ In additional analyses, the authors were able to identify 2 clusters: (1) a cluster of SNPs mainly affecting SHBG levels, with secondary directionally opposing effects on testosterone and (2) a cluster of SNPs affecting testosterone levels, independent of SHBG. To account for this genetic correlation and to explore the independent effects of each exposure, we used 2 different methods. First, we performed cluster-specific MR analyses. Second, we performed multivariable MR analyses. Here, the SNPs associated with BioT and SHBG are combined, and the direct causal effect on the risk of aSAH is estimated for each exposure while accounting for the other exposure.^20^ Also, since the SHBG SNPs were all identified after adjusting for body mass index in the exposure GWAS, we performed sensitivity analysis using the same SNPs with their body mass index unadjusted effects on SHBG.

We calculated all effect estimates as odds ratios (OR) with 95% CIs. For AAM and age at natural menopause, we calculated ORs for aSAH per 1-year increment. For all other exposures, we calculated ORs per 1-SD increase in exposure levels. We estimated 1-SD unit using the estimate_trait_sd() function in the TwoSampleMR package, which estimates the trait SD via obtaining the beta estimates from z-scores and finding the ratio with original β values. We set the *P* value for statistical significance at *P*<0.01, after correcting for multiple exposures using the Bonferroni method (α=0.05/5 exposures). We considered results with *P* values between 0.05 and 0.01 suggestive. We performed all MR analyses using the TwoSampleMR (version 0.5.6), Mendelian randomization (version 0.5.1), and MRPRESSO (version 1.0) packages for R.^18,21,22^

## Results

The primary univariable MR analysis showed an 18% increased aSAH risk among women per 1-SD increase in genetically determined SHBG levels (OR, 1.18 [95% CI, 1.05–1.34]; *P*=0.007) as shown in the Figure. We also found suggestive evidence of a 27% decrease in aSAH risk among women per 1-SD increase in genetically determined BioT (OR, 0.73 [95% CI, 0.55–0.95]; *P*=0.02). However, when only using SNPs from the cluster of BioT-associated SNPs independent of SHBG, the latter association disappeared (17 SNPs; OR, 1.24 [95% CI, 0.81–1.91]; *P*=0.313), as shown in Table S8. The MR-estimate after cluster filtering using SNPs with primary SHBG effects and secondary (opposite) effects on testosterone remained significant (126 SNPs; OR, 1.21 [95% CI, 1.05–1.40]; *P*=0.008). Multivariable MR analyses, however, did not support a direct causal effect of either SHBG (OR, 1.17 [95% CI, 0.94–1.46]; *P*=0.169) or BioT (OR, 1.02 [95% CI, 0.61–1.69]; *P*=0.952) on aSAH risk among women (Table S9). However, the multivariable OR estimate for SHBG was almost identical to the univariable estimate, suggesting low power in the multivariable MR analysis. No associations were observed between genetically determined AAM, age at menopause or estradiol levels, and risk of aSAH (Figure and Table S10). MR estimates were similar between body mass index adjusted and unadjusted SHBG, suggesting no body mass index-related collider bias (Table S10).

**Figure.**
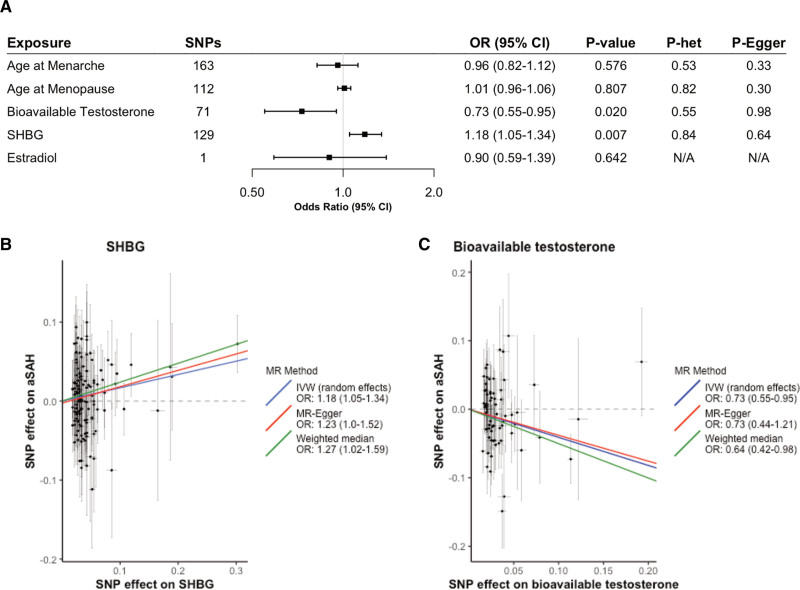
**Effect estimates among women. A**, Inverse-variance weighted estimates for the association between a genetically determined unit increase in exposure on the risk of aneurysmal subarachnoid hemorrhage (aSAH). **B** and **C**, Scatter plots of individual single-nucleotide polymorphism (SNP) effects and estimates from different Mendelian randomization (MR) methods for the effect of (**B**) sex hormone-binding globulin (SHBG) on aSAH and (**C**) bioavailable testosterone on aSAH. *P* het is the *P* value belonging to the Q statistic for heterogeneity. *P* Egger is the *P* value belonging to the Egger intercept. IVW indicates inverse-variance weighted; and OR, odds ratio.

Cochran Q statistic showed no evidence for heterogeneity. The MR-Pleiotropy Residual Sum and Outlier global test and MR-Egger intercepts showed no indication for directional pleiotropic effects. No associations were identified among men (Table S11). MR analyses based on the weighted median method yielded results in the same direction as in the inverse-variance weighted-method, but with lower precision. MR-Steiger filtering was performed for the exposures AAM, age at menopause, SHBG, and BioT to remove SNPs suggestive of reverse causation. Subsequent MR analyses excluding these variants showed comparable results to the main analyses (Table S12).

## Discussion

We showed that genetic predisposition to elevated levels of SHBG, with secondary lower levels of BioT, is associated with an increased risk of aSAH among women. Additionally, we found that genetically determined increased AAM or age at menopause is unlikely to have a substantial effect on aSAH risk. Moreover, associations were only found among women, suggesting a smaller role, if any, of sex hormone levels on aSAH risk among men.

Although SHBG has not been investigated before in the context of aSAH, observational studies implicate a role for SHBG in various vascular diseases. For example, serum SHBG levels are inversely associated with the risk of ischemic stroke in postmenopausal women, even after adjusting for potential mediators such as estradiol, testosterone, and diabetes.^23^ Similarly, higher serum SHBG levels have been associated with optimal cardiovascular health among postmenopausal women.^24^ A previous MR study has also demonstrated a potential causal association between increasing SHBG levels and decreasing risk of coronary heart disease.^25^ However, the mechanism of how SHBG directly affects vascular diseases is currently poorly understood.^26^ Despite the usually protective associations between elevated SHBG and vascular diseases, the direction of effect was the opposite in our study. However, almost all SNPs used for the univariate analyses on SHBG had secondary (opposite) effects on BioT levels. Consequently, the cluster analyses including only SNPs affecting SHBG levels, with secondary (opposite) effects on BioT yielded a similar point estimate.

The effect of SHBG on aSAH risk may be mediated via testosterone. In the literature, it is hypothesized that testosterone deficiency may contribute to vascular aging in both men and women via oxidative stress and inflammation, a process known to affect the risk of aSAH.^5,27^ However, this is currently unclear, partly due to the high complexity of both protective and detrimental effects of androgens on the cerebral vasculature, which may differ based on sex, dose, and age, among many other factors.^28^ For example, androgens appear to have proinflammatory effects under basal conditions, but potentially protective anti-inflammatory effects under pathological conditions, such as hypoxia, endotoxin-induced inflammation, or ischemia.^28^ The vascular protective effects of estradiol, and the potential role of estradiol deficiency in increasing the risk of aSAH among postmenopausal women, have been established in far greater detail. Experimental studies have provided evidence of, for example, endothelial dysfunction and inflammation resulting from estrogen deficiency.^6^ Clinical studies, mainly retrospective case-control studies, have focused on many estrogen-related factors, such as age at menopause, use of hormone replacement therapy or oral contraceptives, pregnancy, and hysterectomies.^3,4^ However, these studies are limited by small case numbers, high risk of confounding, and have, in some instances, provided contradicting evidence. Based on our analyses, the AAM or age at menopause is unlikely to have a major effect on aSAH risk. This may reflect a limited effect of estradiol on aSAH risk since menopause, for example, mainly influences estrogens, as opposed to androgens which decline with age shortly after puberty.^27^ However, although our explorative analysis with 1 SNP for estradiol levels resulted in an inconclusive finding, the substantial (fundamental) evidence behind estradiol and aSAH highlights the importance of further investigation. Overall, our understanding of the mechanisms behind the different sex hormones on the progression of intracranial aneurysms toward rupture remains insufficient and needs further exploration.

### Strengths and Limitations

To our knowledge, this is the first MR study on the associations of sex hormones with risk of aSAH. MR studies have benefits over traditional observational studies, for example, by reducing the risk of residual confounding. As such, we were able to provide novel insights that may contribute to a better understanding of the sex difference in aSAH incidence. Also, we used recently published large-scale GWAS-data and performed multiple sensitivity analyses to assess the robustness of our findings.

Our study also has its limitations. First, data were not available for all relevant exposure SNPs in the outcome GWAS, even after searching for potential proxies. A substantial number of exposure SNPs could, therefore, not be used for our MR analyses. Despite affecting the statistical power to detect small effects, we could still include a fair number of SNPs and perform adequate MR analyses. Second, the genetic correlation between SHBG and BioT restricted our ability to estimate their direct effects on aSAH risk. We performed multivariable analyses and cluster analyses to take this into account, despite both having lower power compared to the univariate models. This especially affected the cluster analyses with BioT SNPs independent of SHBG, for which just 17 SNPs could be used. Third, we only used a single SNP related to serum estradiol levels. GWASs of estradiol levels have been complicated by the fluctuations of serum estradiol levels among premenopausal women but also because of difficulties in measuring low serum levels.^11^ However, we think the use of this single SNP is valid for explorative analyses. Particularly because it is located in a biologically relevant gene (CYP19A1) encoding aromatase, the key enzyme for the aromatization of testosterone to form estradiol. Moreover, the SNP has not been associated with testosterone levels, making it suitable to estimate the independent effect of estradiol.^13^ Also, the SNP has been validated against known or suspected estradiol-related traits, such as bone mineral density and insulin sensitivity, as well as estrogen receptor positive breast cancer and endometrial cancer.^13,29^ Finally, MR estimates lifetime effects rather than acute effects. Lifelong exposure typically has a greater effect on an outcome compared with short-term exposure. This is due to the cumulative effects of most exposures on the associated outcome over time.^30^ Effects of acute hormonal changes, for example, via supplementation, might therefore not be captured.

### Conclusions

This MR study provides evidence that a genetically determined increase in serum SHBG levels, with secondary lower BioT levels, is associated with an increased risk of aSAH among women. Our knowledge about this topic is still insufficient, especially about the potential role of androgens. This highlights the need for further (mechanistic) studies on the role of sex hormones on aSAH risk and their potential as an interventional target to lower aSAH incidence.

## Article Information

### Sources of Funding

None.

### Disclosures

Dr Uyttenboogaart received funding from the Dutch Heart Foundation and Health Holland/TKI Public Private partnership program for other research projects not related to the contents of this article. M.K. Bakker has received funding from The Netherlands Cardiovascular Research Initiative: an initiative with support of the Dutch Heart Foundation, CVON2015-08 ERASE. Dr Ruigrok has received funding from the European Research Council (ERC) under the European Union’s Horizon 2020 research and innovation program (grant agreement no. 852173). The other authors report no conflicts.

### Supplemental Material

Data Sources

Tables S1–S12

ISGC Intracranial Aneurysm Working Group Contributors

STROBE-MR Checklist

## Supplementary Material


