# Supplementary material for: Sex Hormones and Risk of Aneurysmal Subarachnoid Hemorrhage: A Mendelian Randomization Study
Source: Stroke. 2022 Jun 2;53(9):2870–5. doi: 10.1161/STROKEAHA.121.038035 (PMC9389934; doi:10.1161/STROKEAHA.121.038035)
Supplement: Supplementary file 1 [file str-53-2870-s001.pdf]

Supplemental Material

Sex hormones and risk of aneurysmal subarachnoid hemorrhage: a Mendelian randomization study

Rob Molenberg BSc, Chris H.L. Thio PhD, Marlien W. Aalbers MD PhD,
Maarten Uyttenboogaart MD PhD, ISGC Intracranial Aneurysm Working Group,
Susanna C. Larsson PhD, Mark K. Bakker MSc, Ynte M. Ruigrok MD PhD,
Harold Snieder PhD, J. Marc C. van Dijk MD PhD

Contents

- **Data sources**
- **Table S1.** SNPs and proxies used for age at menarche
- **Table S2.** SNPs and proxies used for age at menopause
- **Table S3.** SNPs and proxies used for SHBG in men
- **Table S4.** SNPs and proxies used for SHBG in women
- **Table S5.** SNPs and proxies used for bioavailable testosterone in men
- **Table S6.** SNPs and proxies used for bioavailable testosterone in women
- **Table S7.** Study characteristics and SNP effects for estradiol
- **Table S8.** Mendelian randomization results after cluster filtering
- **Table S9.** Multivariable Mendelian randomization results
- **Table S10.** Mendelian randomization results for the different exposures on aSAH risk among women
- **Table S11.** Sensitivity analyses for the different exposures on aSAH risk among men
- **Table S12.** Mendelian randomization results after MR-Steiger filtering
- **ISGC Intracranial Aneurysm Working Group contributors**
- **STROBE-MR checklist**

Data sources

Detailed information (e.g. summary-level GWAS statistics and lists of underlying cohorts) can be obtained via the links provided below.

Exposure data
Summary statistics for the GWASs on *age at menopause* and *age at menarche* can be accessed from the ReproGen website (www.reprogen.org). Summary-level data for the exposures *SHBG* and *Bioavailable testosterone* are available via the supplemental tables belonging to the original article (https://doi.org/10.1038/s41591-020-0751-5).

Outcome data
GWAS summary-level data regarding aneurysmal subarachnoid hemorrhage can be accessed from the Cerebrovascular Disease Knowledge Portal (www.cerebrovascularportal.org).

TABLE S1. SNPs and proxies used for age at menarche

NO.	SNP	CHR	POSITION (B37)	EA/OA	EAF	BETA	SE	P-VALUE	PROXY SNP	PROXY EA/OA	R^2	PROXY EAF
**1**	rs6678140	1	8436802	T/C	0.6731	-0.0269	0.0041	7.54E-11				
**2**	rs141847393	1	27212209	T/C	0.9182	0.0396	0.0071	2.96E-08				
**3**	rs11209331	1	41456689	T/C	0.5713	0.0238	0.0039	9.83E-10				
**4**	rs11210871	1	44029353	C/G	0.2981	0.04	0.0042	3.07E-21	rs3001723	A/G	0.9077	0.3260
**5**	rs643428	1	54728858	T/C	0.5926	-0.0219	0.004	3.21E-08	rs679200	G/A	0.8243	0.5209
**6**	rs7516763	1	65972550	A/C	0.4691	0.0232	0.0038	1.49E-09				
**7**	rs1040070	1	74977870	C/G	0.5654	0.05	0.0039	7.01E-38	rs953567	A/G	0.86	0.5885
**8**	rs11165924	1	98375448	A/G	0.6767	0.0312	0.0041	4.83E-14				
**9**	rs4561063	1	102520898	T/G	0.4614	0.0312	0.0039	7.89E-16				
**10**	rs6661100	1	150758727	T/C	0.0921	0.0471	0.0067	2.18E-12				
**11**	rs2661339	1	163018934	T/G	0.054	0.0534	0.0088	1.34E-09	rs17440397	G/A	0.9833	0.0626
**12**	rs157877	1	165398744	A/G	0.1265	-0.0843	0.0058	2.25E-48				
**13**	rs506589	1	177894287	T/C	0.8029	0.0695	0.0048	1.54E-47				
**14**	rs4951261	1	205717823	A/C	0.6144	0.0269	0.0039	5.36E-12				
**15**	rs7576624	2	625029	T/C	0.8261	-0.0741	0.005	4.98E-50				
**16**	rs10175423	2	42970161	T/C	0.2967	-0.0247	0.0042	4.25E-09				
**17**	rs111567162	2	56588406	A/T	0.1742	0.0675	0.0051	2.49E-40	rs6545574	T/C	0.9217	0.1720
**18**	rs12467441	2	61685826	T/C	0.8739	-0.0408	0.006	8.99E-12	rs34823499	A/G	0.8069	0.8529
**19**	rs2723065	2	65279414	A/G	0.613	-0.0247	0.0039	2.59E-10				
**20**	rs35935052	2	142302503	T/G	0.148	0.0437	0.0054	5.01E-16				
**21**	rs142058842	2	156621725	C/G	0.8297	-0.0681	0.0051	2.42E-40	rs72899095	C/T	1	0.8469
**22**	rs2271758	2	172701157	T/G	0.4106	-0.0214	0.0039	3.96E-08				
**23**	rs842567	2	184291116	A/C	0.7939	-0.034	0.005	9.38E-12	rs1992645	A/G	0.8725	0.7982
**24**	rs10931831	2	199621641	T/C	0.3556	-0.0531	0.004	1.87E-39				

**25**	rs16841867	2	203168235	C/G	0.8845	0.0456	0.006	2.56E-14	rs12464254	T/C	1	0.8598
**26**	rs6735626	2	213403972	A/G	0.4374	0.0215	0.0039	2.96E-08				
**27**	rs73820560	3	1906245	A/C	0.8562	-0.0319	0.0057	1.68E-08				
**28**	rs9867904	3	18442437	C/G	0.3869	-0.0278	0.004	3.42E-12	rs9812165	C/T	1	0.3539
**29**	rs73035994	3	24206463	T/C	0.9716	-0.0907	0.0116	5.22E-15				
**30**	rs1984870	3	24715135	T/G	0.4728	0.0422	0.0039	5.61E-27				
**31**	rs6445624	3	51358019	A/G	0.1473	0.0422	0.0056	6.41E-14	rs4687569	T/C	0.9164	0.1183
**32**	rs7431217	3	68595634	T/C	0.412	0.0229	0.0039	6.19E-09				
**33**	rs9758500	3	86910329	A/G	0.375	-0.0457	0.004	1.36E-30				
**34**	rs10934420	3	117552111	T/C	0.5054	-0.0546	0.0038	1.54E-47				
**35**	rs2461794	3	127870060	A/G	0.2763	0.0342	0.0043	2.10E-15				
**36**	rs6439713	3	137128815	A/C	0.315	0.0259	0.0041	2.13E-10				
**37**	rs11711674	3	156532953	T/C	0.5708	0.0216	0.0038	1.77E-08				
**38**	rs2300922	3	185651469	T/C	0.4144	0.0432	0.0039	1.11E-28				
**39**	rs2108753	4	3266860	T/C	0.565	0.0284	0.0038	1.20E-13				
**40**	rs4340786	4	28746246	A/T	0.7412	0.037	0.0043	9.25E-18	rs10013470	G/T	1	0.7316
**41**	rs3113862	4	95143122	A/G	0.5994	-0.0373	0.0039	9.69E-22				
**42**	rs3733632	4	104640935	A/G	0.8437	-0.0536	0.0052	1.04E-24				
**43**	rs17035311	4	106066293	A/C	0.854	0.036	0.0054	2.26E-11				
**44**	rs13120031	4	177465182	T/C	0.3243	0.0274	0.0041	1.57E-11				
**45**	rs10521021	5	35030311	T/G	0.6577	-0.024	0.0041	4.77E-09				
**46**	rs7712046	5	43134968	T/C	0.6962	-0.0333	0.0042	9.42E-16				
**47**	rs813301	5	52909927	T/C	0.6259	0.0274	0.0039	2.84E-12				
**48**	rs256350	5	59140876	T/C	0.7259	-0.0238	0.0043	2.84E-08				
**49**	rs13173441	5	77048448	T/C	0.8787	0.0331	0.0059	1.86E-08	rs34541757	C/T	0.9912	0.8718
**50**	rs17085593	5	95630705	C/G	0.6841	0.0246	0.0042	3.53E-09	rs2350002	A/G	0.9954	0.6879
**51**	rs654354	5	110503301	A/T	0.3828	-0.0233	0.0039	2.36E-09	rs32701	A/C	1	0.3708
**52**	rs247520	5	110876057	T/C	0.7652	0.0361	0.0045	1.99E-15				

**53**	rs3815212	5	137761555	T/C	0.7829	0.0337	0.0046	3.21E-13				
**54**	rs1428120	5	153541904	T/G	0.5718	0.025	0.0038	7.72E-11				
**55**	rs437836	5	156715068	T/C	0.1671	0.035	0.0052	1.15E-11				
**56**	rs9647570	5	167370263	T/G	0.8544	-0.0363	0.0056	6.26E-11				
**57**	rs6864818	5	168734867	T/C	0.2107	0.0364	0.0046	4.67E-15	rs12716241	T/C	1	0.2256
**58**	rs446745	6	14918298	T/C	0.2407	-0.0258	0.0046	2.81E-08	rs6930513	T/C	0.96	0.2167
**59**	rs6927679	6	18559687	T/C	0.718	0.027	0.0043	2.52E-10				
**60**	rs1539310	6	22562485	A/G	0.7568	0.0244	0.0045	4.59E-08				
**61**	rs9349203	6	41893323	A/G	0.5463	-0.0395	0.0038	5.93E-25				
**62**	rs9474996	6	54640512	A/T	0.5553	-0.0343	0.0038	3.82E-19	rs9474997	T/C	1	0.5845
**63**	rs9382676	6	56859084	T/C	0.7767	0.0373	0.0046	8.77E-16	rs9396246	C/T	0.9946	0.7555
**64**	rs7753896	6	76347020	A/G	0.3681	0.031	0.0039	2.46E-15				
**65**	rs7757654	6	77173780	T/C	0.2956	-0.0306	0.0042	2.51E-13				
**66**	rs9403051	6	100194846	A/G	0.5701	0.0365	0.0038	1.76E-21				
**67**	rs395962	6	105397418	T/G	0.3163	0.1266	0.0041	2.26E-213				
**68**	rs4897178	6	126727908	T/G	0.5545	0.0426	0.0039	1.02E-27	rs4897179	G/A	0.996	0.5457
**69**	rs6911527	6	148285329	T/C	0.2275	0.0269	0.0045	3.18E-09				
**70**	rs6933660	6	151803754	A/C	0.3173	-0.0343	0.0041	8.99E-17				
**71**	rs10268051	7	27763590	A/C	0.7769	0.0249	0.0045	3.88E-08				
**72**	rs17171852	7	41392815	A/C	0.8078	-0.038	0.0048	3.42E-15				
**73**	rs1470750	7	50576648	C/G	0.5923	-0.0223	0.0039	1.44E-08	rs12718572	C/T	0.9287	0.6431
**74**	rs2267812	7	74138121	A/C	0.7951	0.0417	0.0049	1.69E-17	rs4717907	G/A	0.8269	0.73613
**75**	rs1030015	7	78139581	T/G	0.5217	-0.0206	0.0038	4.45E-08				
**76**	rs149226155	7	93215658	A/G	0.3474	-0.0238	0.0041	5.67E-09	rs13247665	C/T	0.9912	0.35608
**77**	rs999885	7	99701176	A/G	0.5164	0.0241	0.0038	2.79E-10				
**78**	rs11767400	7	122160742	A/C	0.2959	0.0289	0.0042	5.41E-12				
**79**	rs12707076	7	132729814	C/G	0.3844	0.0273	0.004	4.88E-12	rs6953845	C/T	1	0.3936
**80**	rs7004265	8	1523903	T/C	0.4769	0.023	0.0039	5.12E-09	rs12546094	T/C	0.9297	0.4851

**81**	rs2724961	8	4560227	T/C	0.4682	-0.0459	0.0038	3.76E-33				
**82**	rs6185	8	25280800	C/G	0.7296	-0.0301	0.0044	9.48E-12	rs4871939	G/A	0.9787	0.7535
**83**	rs16918378	8	53877882	T/C	0.8769	0.0478	0.0059	9.08E-16				
**84**	rs11786868	8	77653945	C/G	0.837	0.0321	0.0051	4.27E-10	rs7837649	G/A	0.993	0.8310
**85**	rs10094506	8	78116203	T/C	0.2807	-0.0454	0.0043	2.46E-26	rs72663709	T/C	1	0.2913
**86**	rs7465046	8	87319950	T/C	0.2324	-0.0409	0.0046	1.52E-18	rs4464946	A/G	0.9845	0.2594
**87**	rs2441873	8	105329549	T/G	0.4106	0.0235	0.0039	1.74E-09	rs2514656	G/A	0.9959	0.4185
**88**	rs2542420	8	140645701	C/G	0.5384	0.0327	0.004	1.52E-16	rs2615377	T/C	0.9566	0.5348
**89**	rs552491	9	1711210	A/G	0.6375	-0.0294	0.004	1.11E-13				
**90**	rs1601615	9	11813745	T/C	0.3964	-0.028	0.0039	7.57E-13				
**91**	rs7849973	9	22819576	C/G	0.6547	0.024	0.004	2.82E-09	rs4571809	T/G	0.9203	0.6412
**92**	rs1329767	9	73798371	A/C	0.3487	-0.0292	0.004	2.43E-13				
**93**	rs1571536	9	92215638	T/C	0.4852	0.0331	0.0038	2.19E-18				
**94**	rs9330454	9	92515514	A/G	0.4299	-0.0307	0.0042	1.44E-13	rs9286380	T/C	0.8525	0.4583
**95**	rs10156597	9	108941509	A/T	0.6774	0.1024	0.0041	5.04E-139	rs13286861	C/T	0.9955	0.6730
**96**	rs7852169	9	114318394	C/G	0.9117	-0.0973	0.0068	1.82E-46	rs7040225	C/T	1	0.9205
**97**	rs2780243	9	120730928	T/C	0.5646	-0.0234	0.0039	2.31E-09				
**98**	rs7912468	10	2697434	T/C	0.5775	-0.0239	0.0039	1.20E-09	rs10751883	C/T	0.9447	0.5656
**99**	rs10906395	10	13541008	T/C	0.6109	-0.0233	0.0039	2.28E-09				
**100**	rs61846901	10	51056858	T/C	0.3113	-0.0257	0.0042	1.21E-09	rs11599257	G/A	0.991	0.3280
**101**	rs6415872	10	63660689	A/G	0.4931	0.0236	0.0039	1.52E-09				
**102**	rs4746113	10	74071178	A/G	0.3094	-0.0244	0.0042	8.79E-09				
**103**	rs77532868	10	88081438	T/C	0.0446	0.0573	0.0097	3.31E-09				
**104**	rs2066323	10	104871361	A/G	0.6021	-0.0237	0.0039	1.31E-09				
**105**	rs10400136	10	120833948	A/G	0.5627	-0.0259	0.0039	2.73E-11	rs11198754	G/A	0.8804	0.6054
**106**	rs12571664	10	121708929	T/C	0.7998	0.0367	0.0048	1.85E-14	rs11199129	A/G	0.9117	0.7505
**107**	rs7077302	10	123676662	C/G	0.0852	0.0497	0.007	1.08E-12	rs72839625	A/C	0.8108	0.0845
**108**	rs4576738	10	134294398	A/G	0.4455	0.0266	0.0043	7.20E-10	rs7904728	A/G	0.9184	0.4811

**109**	rs3782120	11	206089	A/G	0.2583	0.0334	0.0044	3.69E-14	rs2293167	G/A	0.975	0.2694
**110**	rs10832021	11	13324530	A/G	0.7095	-0.047	0.0042	6.27E-29				
**111**	rs4359170	11	16596152	A/T	0.673	0.0282	0.0041	5.99E-12	rs11023987	T/C	1	0.6610
**112**	rs11606190	11	28033473	A/G	0.1457	0.041	0.0056	1.61E-13				
**113**	rs11031040	11	30317733	T/G	0.8378	-0.0404	0.0052	5.10E-15				
**114**	rs1023955	11	43608835	T/G	0.3976	-0.0279	0.0039	7.36E-13				
**115**	rs10897450	11	63593219	C/G	0.5304	0.0229	0.0038	2.18E-09	rs626333	A/G	0.9921	0.4960
**116**	rs7115444	11	77555824	T/C	0.2085	0.0333	0.0048	3.82E-12				
**117**	rs4402316	11	84780098	C/G	0.2407	0.0313	0.0047	3.22E-11	rs1826613	T/C	0.9662	0.2863
**118**	rs6590889	11	101438191	T/C	0.3404	-0.0437	0.004	8.62E-28				
**119**	rs7114175	11	122813983	A/T	0.4963	-0.0599	0.0038	1.14E-56	rs7110373	C/T	0.996	0.5070
**120**	rs7132908	12	50263148	A/G	0.3883	-0.0424	0.0039	6.64E-27				
**121**	rs1148006	12	75978358	A/G	0.2442	-0.0259	0.0044	5.81E-09	rs1148005	A/G	0.9535	0.2922
**122**	rs7979001	12	97506357	A/G	0.5078	0.0219	0.0038	6.13E-09				
**123**	rs660549	12	121300988	T/C	0.5671	-0.0212	0.0038	3.36E-08				
**124**	rs9548873	13	40238492	T/C	0.663	-0.0311	0.004	7.50E-15				
**125**	rs9568123	13	49475780	A/G	0.8478	-0.0291	0.0053	4.06E-08				
**126**	rs1925047	13	74600274	A/C	0.3213	-0.0341	0.0041	5.70E-17	rs1570739	C/T	0.94	0.3052
**127**	rs11619721	13	112082513	T/G	0.0836	-0.0413	0.0073	1.33E-08				
**128**	rs9522262	13	112186283	C/G	0.4915	0.0411	0.0039	1.69E-25	rs2026174	C/T	0.9841	0.4781
**129**	rs10136330	14	30514335	T/C	0.0426	-0.0579	0.01	6.65E-09				
**130**	rs10138913	14	60943106	T/C	0.3062	0.056	0.0041	1.14E-41				
**131**	rs941520	14	99709702	A/C	0.4936	-0.0223	0.0039	8.99E-09				
**132**	rs12894936	14	100846991	T/C	0.2936	-0.0522	0.0043	3.73E-34	rs2273800	A/G	0.8053	0.2734
**133**	rs4924538	15	41494364	A/T	0.5129	-0.0255	0.0042	1.03E-09	rs11855102	C/T	0.8014	0.4503
**134**	rs1435753	15	47925066	T/C	0.6467	-0.0278	0.004	2.91E-12				
**135**	rs3743266	15	60781513	T/C	0.668	0.0416	0.004	8.52E-25				
**136**	rs72756954	15	64537300	C/G	0.938	0.0577	0.0081	8.45E-13	rs72756953	C/A	1	0.9384

**137**	rs5742915	15	74336633	T/C	0.5463	-0.0234	0.0038	1.02E-09				
**138**	rs1971554	15	83406228	T/C	0.2654	0.032	0.0044	2.35E-13				
**139**	rs12915845	15	89042467	T/C	0.4244	-0.0403	0.0039	3.70E-25				
**140**	rs153793	16	15542199	A/G	0.5198	-0.0234	0.0038	9.27E-10	rs222132	G/A	1	0.5477
**141**	rs112991346	16	19967668	T/C	0.8575	-0.0446	0.0055	6.47E-16	rs11641335	C/T	1	0.8688
**142**	rs143461173	16	52283158	A/G	0.8063	0.0288	0.005	6.10E-09	rs72794118	A/G	1	0.8211
**143**	rs9972653	16	53814363	T/G	0.4002	-0.0509	0.0039	6.47E-40				
**144**	rs7359336	16	69733460	A/G	0.5789	-0.0534	0.0038	5.33E-44				
**145**	rs142643995	17	2017993	T/C	0.0302	0.0646	0.0117	3.25E-08				
**146**	rs12603280	17	6034754	A/G	0.244	-0.037	0.0045	2.66E-16	rs7215990	A/G	0.9524	0.2465
**147**	rs55680968	17	7774047	A/G	0.9282	-0.0455	0.0075	1.17E-09				
**148**	rs9635759	17	49613785	A/G	0.3067	0.059	0.0041	2.78E-46	rs9635758	A/G	0.9857	0.2962
**149**	rs2787487	17	53209382	C/G	0.6037	0.0311	0.0039	1.62E-15	rs244340	A/G	0.9837	0.5815
**150**	rs66508321	17	78739672	A/G	0.3235	-0.0303	0.0041	2.84E-13				
**151**	rs8087304	18	31765736	A/T	0.4823	0.0222	0.0038	6.54E-09	rs11081846	A/G	0.9842	0.4791
**152**	rs1512238	18	44748467	A/G	0.4207	-0.0537	0.0038	2.48E-44				
**153**	rs484353	19	7891767	A/G	0.5389	0.0316	0.0039	8.28E-16				
**154**	rs4804117	19	9984509	T/G	0.5568	0.0455	0.0039	3.58E-31	rs8110445	C/T	0.9763	0.5378
**155**	rs4804025	19	47609223	A/G	0.7038	-0.0409	0.0042	3.11E-22	rs2303108	C/T	0.9816	0.6909
**156**	rs2548458	19	49209325	T/C	0.5061	0.0212	0.0038	3.53E-08	rs507766	C/T	0.9762	0.4682
**157**	rs852061	20	17109159	A/C	0.3645	-0.0365	0.0039	1.58E-20				
**158**	rs1535252	20	19682834	T/C	0.4547	-0.0252	0.0038	4.82E-11				
**159**	rs2295094	20	33447915	A/G	0.1585	0.0364	0.0053	5.86E-12				
**160**	rs36093651	20	37287102	T/C	0.2378	0.0371	0.0046	6.51E-16				
**161**	rs3746619	20	54823805	A/C	0.0862	0.0475	0.0069	5.52E-12				
**162**	rs151680	22	22273242	T/C	0.524	0.028	0.0038	2.52E-13				
**163**	rs9614460	22	45745229	T/G	0.6779	-0.0249	0.0041	1.38E-09				

SNP, single nucleotide polymorphism; Chr, chromosome; EA, effect allele; OA, other allele; EAF, effect allele frequency; SE, standard error.

TABLE S2. SNPs and proxies used for age at menopause

NO.	SNP	CHR	POSITION (B37)	EA/OA	EAF	BETA	SE	P-VALUE	PROXY SNP	PROXY EA/OA	R^2	PROXY EAF
**1**	rs9438982	1	39358143	C/A	0.3219	0.214	0.013	6.40E-62	rs9438979	G/T	0.9955	0.328
**2**	rs12046563	1	43137280	A/G	0.7609	0.1	0.014	1.70E-12				
**3**	rs12133213	1	110191395	G/A	0.4657	0.08	0.013	5.30E-10	rs542338	A/G	0.9521	0.4105
**4**	rs72708144	1	149815740	C/T	0.0404	0.282	0.032	1.10E-18	rs41265172	T/C	0.8968	0.0378
**5**	rs2736609	1	156202640	T/C	0.3592	0.079	0.013	3.00E-10				
**6**	rs11582336	1	178230945	A/G	0.7951	0.106	0.015	2.00E-12				
**7**	rs1044595	1	180943529	T/C	0.5979	0.132	0.012	1.30E-26	rs12754041	G/T	0.9639	0.5686
**8**	rs7515939	1	225626327	A/T	0.3336	0.103	0.013	8.40E-16	rs6702538	A/C	1	0.3588
**9**	rs7414807	1	231476874	A/G	0.6326	0.088	0.013	4.00E-12				
**10**	rs7539755	1	244598815	C/T	0.5766	0.101	0.012	1.40E-16	rs6684936	T/C	0.9919	0.5646
**11**	rs7779	1	246930564	C/G	0.0733	0.148	0.024	3.90E-10	rs72764632	T/C	0.984	0.0656
**12**	rs780088	2	27716004	T/C	0.606	0.2	0.012	1.20E-58				
**13**	rs12053063	2	28350611	G/A	0.7212	0.103	0.014	2.40E-14	rs1948922	A/G	0.964	0.7147
**14**	rs17425341	2	44324287	T/G	0.6567	0.085	0.013	2.30E-11				
**15**	rs76928871	2	48005821	G/A	0.1896	0.198	0.016	2.10E-37				
**16**	rs62156756	2	67671015	A/G	0.8963	0.263	0.02	2.20E-40				
**17**	rs4852777	2	71534161	G/C	0.404	0.074	0.012	2.10E-09	rs4852778	G/A	0.926	0.4443
**18**	rs6727266	2	112007716	G/A	0.5057	0.079	0.012	9.90E-11				
**19**	rs16830019	2	152267359	T/G	0.2626	0.118	0.014	2.10E-17				
**20**	rs4668354	2	171814750	G/C	0.6262	0.187	0.012	5.50E-51	rs6731774	T/C	0.9957	0.6402
**21**	rs72934556	2	203990789	G/T	0.1258	0.101	0.018	3.70E-08				
**22**	rs6736096	2	216958369	C/T	0.5235	0.098	0.012	3.10E-16				
**23**	rs7558434	2	223705337	T/G	0.4388	0.07	0.012	9.10E-09				
**24**	rs12636454	3	12360214	C/T	0.2451	0.092	0.014	6.90E-11				

**25**	rs62244773	3	30518433	A/T	0.6987	0.083	0.014	1.80E-09	rs62244772	T/C	0.8484	0.6581
**26**	rs12487736	3	47459679	C/T	0.4205	0.078	0.012	3.50E-10	rs6792461	T/C	0.936	0.3936
**27**	rs9968117	3	52838654	T/C	0.1188	0.106	0.019	1.80E-08				
**28**	rs2885255	3	57722613	C/G	0.5976	0.071	0.012	8.60E-09	rs7624585	T/C	1	0.6024
**29**	rs7610102	3	101246803	G/A	0.6052	0.077	0.013	1.30E-09	rs7634759	C/A	1	0.5915
**30**	rs6793835	3	135819934	G/A	0.7383	0.141	0.014	1.80E-24				
**31**	rs10154963	3	150169559	C/T	0.2798	0.077	0.014	1.80E-08				
**32**	rs10804920	3	189438689	T/C	0.4389	0.084	0.012	6.00E-12				
**33**	rs13070791	3	193385275	T/C	0.433	0.074	0.012	1.40E-09				
**34**	rs2052160	4	13576578	C/G	0.8928	0.168	0.02	1.90E-17	rs7661090	C/T	0.9905	0.8817
**35**	rs6824237	4	38071911	T/C	0.7506	0.077	0.014	3.50E-08				
**36**	rs76540949	4	48527209	T/C	0.5221	0.141	0.012	9.60E-31	rs12512637	A/G	0.8693	0.4811
**37**	rs12651246	4	84367605	A/G	0.4875	0.261	0.012	2.20E-103				
**38**	rs6810489	4	99877445	T/G	0.5958	0.09	0.012	4.40E-13				
**39**	rs9990489	4	188916240	C/T	0.5899	0.098	0.013	8.50E-15				
**40**	rs274701	5	6728707	C/A	0.5921	0.144	0.012	3.20E-31				
**41**	rs62356073	5	36241922	G/A	0.4264	0.07	0.012	1.60E-08				
**42**	rs17206591	5	51997134	A/C	0.5634	0.074	0.012	1.40E-09				
**43**	rs7728833	5	82000737	A/G	0.4997	0.079	0.012	5.10E-11				
**44**	rs10070308	5	107281621	C/T	0.844	0.099	0.017	2.80E-09				
**45**	rs10477172	5	141682090	T/C	0.4883	0.092	0.012	2.80E-14				
**46**	rs888694	5	154307485	A/C	0.0862	0.16	0.022	1.30E-13	rs2688187	G/A	0.99	0.1123
**47**	rs353478	5	176370988	C/T	0.4855	0.318	0.012	4.20E-154				
**48**	rs9348724	6	10887276	C/G	0.1724	0.306	0.016	6.90E-80	rs9379896	C/T	0.9037	0.1849
**49**	rs113967617	6	111663858	A/G	0.1544	0.122	0.017	5.70E-13				
**50**	rs6569648	6	130349119	C/T	0.2358	0.123	0.014	6.90E-18				
**51**	rs11767307	7	23584496	C/G	0.7697	0.084	0.014	4.90E-09	rs7799435	C/T	1	0.7773
**52**	rs10255049	7	56121304	G/A	0.3121	0.14	0.013	1.00E-26				

**53**	rs2056726	7	99780283	G/A	0.7803	0.107	0.015	3.40E-13				
**54**	rs2392836	7	105973193	G/A	0.3726	0.115	0.013	4.00E-20				
**55**	rs4731541	7	128678236	C/G	0.3733	0.099	0.013	2.40E-15	rs7796963	T/C	0.9788	0.3678
**56**	rs2013	7	158523888	C/T	0.1659	0.091	0.017	4.90E-08				
**57**	rs3735828	8	61592425	G/A	0.3594	0.115	0.013	4.80E-19	rs1017861	A/G	0.8578	0.341
**58**	rs1467044	8	120887041	G/A	0.4597	0.07	0.012	9.70E-09				
**59**	rs6470598	8	129018915	C/T	0.208	0.085	0.015	1.00E-08	rs6984736	A/G	0.9574	0.163
**60**	rs1476164	8	129620616	A/T	0.7837	0.094	0.015	1.30E-10	rs73707884	T/G	1	0.7823
**61**	rs4879656	9	33012382	C/A	0.6248	0.133	0.013	1.90E-26				
**62**	rs10818873	9	126559993	C/T	0.0647	0.218	0.025	1.20E-18				
**63**	rs74701710	10	13208912	G/A	0.9436	0.216	0.027	1.20E-15				
**64**	rs10764106	10	37055384	C/T	0.5273	0.078	0.012	1.60E-10				
**65**	rs10823203	10	70219610	G/C	0.7924	0.159	0.015	2.90E-26	rs10823204	T/C	1	0.7903
**66**	rs7087644	10	97826334	A/G	0.9576	0.289	0.031	3.20E-21				
**67**	rs7091889	10	104793723	G/A	0.214	0.121	0.015	2.80E-16				
**68**	rs728900	10	131590300	T/A	0.5771	0.144	0.013	6.90E-31	rs4750773	G/A	0.8658	0.5875
**69**	rs7928823	11	9475372	A/G	0.4285	0.069	0.012	2.20E-08	rs7928945	A/G	0.984	0.4473
**70**	rs11031006	11	30226528	A/G	0.146	0.201	0.017	1.50E-31				
**71**	rs10899493	11	78117534	C/T	0.1664	0.12	0.016	1.90E-13				
**72**	rs10743724	12	30776022	C/T	0.5197	0.076	0.012	3.50E-10				
**73**	rs2277339	12	57146069	T/G	0.8941	0.37	0.021	4.60E-68				
**74**	rs7308068	12	66696706	C/T	0.4705	0.119	0.012	2.90E-22				
**75**	rs12825762	12	122983973	G/A	0.4495	0.09	0.012	1.90E-13				
**76**	rs35067339	12	123760109	T/A	0.7961	0.178	0.015	7.40E-32	rs6633	G/T	0.9943	0.7773
**77**	rs7318091	13	31203339	C/T	0.5954	0.068	0.012	4.10E-08				
**78**	rs3736830	13	50306221	C/G	0.8453	0.116	0.017	4.00E-12	rs9535306	G/A	1	0.829
**79**	rs7322160	13	61061456	C/T	0.3325	0.186	0.013	7.20E-48				
**80**	rs12879626	14	34721134	T/G	0.3876	0.093	0.013	1.00E-13				

**81**	rs61488898	14	35086437	C/T	0.9738	0.356	0.04	2.70E-19				
**82**	rs1969713	14	45623460	C/T	0.1044	0.168	0.02	5.30E-17				
**83**	rs762643	14	54422767	G/T	0.5561	0.076	0.012	6.20E-10				
**84**	rs1986616	14	73540936	G/A	0.4773	0.08	0.012	4.30E-11				
**85**	rs9796	15	41271447	A/T	0.5351	0.181	0.012	1.90E-49	rs2306083	G/A	0.8434	0.5209
**86**	rs11071756	15	63831636	C/G	0.8603	0.12	0.017	4.90E-12	rs289818	A/G	0.9825	0.8698
**87**	rs716886	15	83715853	A/G	0.3294	0.084	0.013	7.10E-11				
**88**	rs12898357	15	89801391	A/G	0.6145	0.183	0.013	1.50E-48				
**89**	rs9673473	16	12011212	C/G	0.4029	0.212	0.012	3.40E-66	rs28653099	C/A	0.996	0.4503
**90**	rs11075466	16	52375540	G/A	0.1764	0.095	0.016	4.10E-09				
**91**	rs8045027	16	79388209	G/A	0.4644	0.073	0.012	2.50E-09				
**92**	rs200293726	16	79754440	T/A	0.3098	0.106	0.016	1.60E-11	rs4575545	A/G	0.9911	0.336
**93**	rs2108839	16	89860182	T/G	0.6903	0.136	0.013	6.90E-25	rs6500452	T/C	1	0.6829
**94**	rs34856659	17	5327572	C/T	0.2431	0.168	0.014	7.80E-33				
**95**	rs111637825	17	40134782	G/A	0.9357	0.139	0.025	1.90E-08				
**96**	rs1815198	17	55360585	A/G	0.3564	0.09	0.013	1.30E-12	rs13353194	G/A	0.9701	0.3598
**97**	rs34609096	17	71230680	A/G	0.4696	0.068	0.012	2.30E-08	rs1566286	G/A	0.9879	0.4423
**98**	rs12605881	18	60941441	T/A	0.4055	0.07	0.012	2.00E-08	rs12968517	T/C	0.9127	0.3857
**99**	rs11670032	19	23166913	T/C	0.8557	0.182	0.017	1.60E-25				
**100**	rs424223	19	23485893	T/G	0.2633	0.1	0.014	4.60E-13	rs295353	C/T	1	0.2495
**101**	rs7249357	19	33465388	G/A	0.811	0.136	0.016	1.40E-18				
**102**	rs11668344	19	55833664	A/G	0.6403	0.447	0.013	1.00E-200				
**103**	rs236117	20	5937175	G/T	0.8899	0.225	0.019	1.00E-31				
**104**	rs58065489	20	32052912	C/G	0.8292	0.098	0.016	1.90E-09	rs112523421	C/T	1	0.839
**105**	rs11699793	20	34271574	C/T	0.8972	0.123	0.02	7.00E-10				
**106**	rs483508	20	48499609	T/C	0.3847	0.117	0.013	6.80E-21				
**107**	rs7266248	20	55183547	G/A	0.1986	0.115	0.015	3.60E-14				
**108**	rs10854167	20	61533039	G/C	0.7893	0.175	0.015	1.40E-32	rs2295000	G/A	0.9809	0.8121

**109**	rs9975728	21	40662749	T/G	0.8711	0.104	0.018	7.50E-09				
**110**	rs5754100	22	21916166	C/T	0.1906	0.097	0.016	1.90E-09	rs5754217	T/G	0.9122	0.1938
**111**	rs5762852	22	29242473	C/T	0.1545	0.131	0.017	5.80E-15				
**112**	rs2272805	22	45809698	G/A	0.8545	0.108	0.017	4.30E-10				

SNP, single nucleotide polymorphism; Chr, chromosome; EA, effect allele; OA, other allele; EAF, effect allele frequency; SE, standard error.

Table S3. SNPs and proxies used for SHBG in men

NO.	SNP	CHR	POSITION (B37)	EA/OA	EAF	BETA	SE	P-VALUE	PROXY SNP	PROXY EA/OA	R^2	PROXY EAF
**1**	rs36086195	1	16510894	T/C	0.579	0.017	0.001	2.3E-52	rs6656611	C/T	0.8302	0.5835
**2**	rs59708846	1	61687651	A/G	0.076	0.019	0.002	1.3E-20	rs17311684	G/A	0.8409	0.0686
**3**	rs1730865	1	107605611	G/T	0.345	0.028	0.001	8.0E-127	rs3108680	C/T	0.9513	0.331
**4**	rs12059956	1	171063262	G/A	0.583	0.007	0.001	3.4E-10				
**5**	rs17583875	1	197924770	A/G	0.021	0.023	0.004	1.1E-09	rs115328872	A/G	1	0.0219
**6**	rs10864086	1	214318748	C/A	0.256	0.015	0.001	2.9E-32				
**7**	rs2820441	1	219734960	C/A	0.318	0.009	0.001	2.7E-13				
**8**	rs2247213	1	221055463	G/A	0.670	0.015	0.001	1.3E-36				
**9**	rs1870927	1	226426337	A/T	0.620	0.007	0.001	2.3E-08	rs6605032	T/C	0.8303	0.6799
**10**	rs144647926	1	235467607	A/G	0.087	0.013	0.002	1.3E-10				
**11**	rs1260326	2	27730940	C/T	0.606	0.041	0.001	1.3E-298				
**12**	rs17050272	2	121306440	A/G	0.410	0.007	0.001	1.4E-10	rs6706968	A/C	0.9796	0.4592
**13**	rs13389219	2	165528876	T/C	0.394	0.012	0.001	2.3E-26				
**14**	rs72948115	2	178167086	C/T	0.905	0.012	0.002	2.2E-10				
**15**	rs8176526	2	188345322	C/T	0.730	0.008	0.001	1.6E-10				
**16**	rs4675682	2	208402750	T/C	0.540	0.009	0.001	9.2E-16				
**17**	rs62182125	2	219274142	G/A	0.449	0.008	0.001	1.2E-12	rs2303561	T/C	0.855	0.4066
**18**	rs12694450	2	220019638	T/C	0.325	0.007	0.001	3.0E-10	rs6725951	T/C	0.9957	0.3579
**19**	rs2222018	2	227095220	C/A	0.352	0.014	0.001	5.1E-31				
**20**	rs10153800	2	242179134	A/G	0.230	0.007	0.001	2.9E-08				
**21**	rs17036326	3	12389313	G/A	0.122	0.019	0.002	1.2E-26				
**22**	rs2564923	3	53103262	A/G	0.443	0.006	0.001	8.1E-11				
**23**	rs13315174	3	105406468	G/A	0.784	0.008	0.001	1.5E-10	rs4627779	G/A	0.893	0.7624
**24**	rs687339	3	135932359	C/T	0.228	0.025	0.001	5.3E-84	rs6779146	C/T	0.9454	0.2356
**25**	rs234051	3	172311712	A/G	0.284	0.009	0.001	1.8E-15				

**26**	rs7631981	3	185273510	G/A	0.697	0.008	0.001	6.5E-13				
**27**	rs11734408	4	23882519	G/A	0.293	0.008	0.001	2.1E-13				
**28**	rs28507491	4	77197651	A/G	0.376	0.014	0.001	2.0E-31	rs28578510	T/C	1	0.3897
**29**	rs7694379	4	88186509	G/A	0.567	0.020	0.001	5.5E-71				
**30**	rs6831352	4	100063525	T/C	0.302	0.020	0.001	2.9E-58				
**31**	rs7655064	4	120106348	T/C	0.875	0.008	0.002	4.0E-08				
**32**	rs10027275	4	148981496	G/C	0.259	0.013	0.001	3.9E-26	rs12650033	G/A	0.9504	0.2664
**33**	rs72729610	4	154190965	A/G	0.834	0.009	0.002	9.1E-11				
**34**	rs11732763	4	171010101	A/G	0.106	0.011	0.002	1.9E-10				
**35**	rs7735249	5	53310139	C/G	0.887	0.020	0.002	4.9E-31	rs112838464	G/A	1	0.8857
**36**	rs40270	5	55804552	A/C	0.228	0.014	0.001	4.0E-26				
**37**	rs11739158	5	72927292	T/C	0.428	0.008	0.001	1.7E-13				
**38**	rs6595447	5	122750847	T/C	0.807	0.010	0.001	1.2E-12				
**39**	rs329122	5	133864599	G/A	0.581	0.008	0.001	1.5E-14				
**40**	rs11743810	5	137802404	T/C	0.560	0.008	0.001	3.6E-11				
**41**	rs2431752	5	162882702	A/G	0.106	0.011	0.002	7.5E-10				
**42**	rs55646464	5	173324971	G/T	0.700	0.009	0.001	1.3E-11				
**43**	rs17185536	6	100620931	T/C	0.244	0.007	0.001	3.2E-09				
**44**	rs1890426	6	116338065	C/T	0.401	0.007	0.001	1.8E-09				
**45**	rs6900473	6	130375810	A/G	0.312	0.010	0.001	4.8E-16				
**46**	rs501470	6	160770918	G/T	0.475	0.017	0.001	3.4E-55				
**47**	rs2106727	7	17287998	G/A	0.637	0.006	0.001	2.8E-08				
**48**	rs860262	7	28194397	A/C	0.502	0.011	0.001	3.1E-23				
**49**	rs6965401	7	46269012	G/A	0.936	0.015	0.002	9.0E-10				
**50**	rs12536766	7	70158864	T/G	0.570	0.007	0.001	5.9E-09				
**51**	rs17145750	7	73026378	T/C	0.161	0.013	0.002	1.4E-16				
**52**	rs1229492	7	81564122	T/C	0.268	0.011	0.001	1.2E-18				
**53**	rs445	7	92408370	C/T	0.905	0.013	0.002	7.9E-12				

**54**	rs6950023	7	97915635	G/T	0.814	0.030	0.001	2.1E-101	rs112758337	G/A	0.9417	0.8181
**55**	rs187437	7	116445091	G/A	0.547	0.009	0.001	3.2E-17				
**56**	rs157935	7	130585553	G/T	0.304	0.012	0.001	5.0E-27				
**57**	rs3812275	7	135064882	C/A	0.403	0.007	0.001	9.6E-09				
**58**	rs114949263	7	150498245	C/T	0.111	0.017	0.002	9.1E-23				
**59**	rs12543287	8	42334511	C/G	0.371	0.010	0.001	2.9E-21	rs10808961	A/G	0.8247	0.337
**60**	rs10107182	8	59392737	T/C	0.663	0.013	0.001	1.1E-27				
**61**	rs75349541	8	71152803	C/T	0.867	0.009	0.002	2.0E-08				
**62**	rs11994858	8	81273210	G/A	0.654	0.011	0.001	4.4E-23				
**63**	rs10116426	9	4145648	C/A	0.432	0.008	0.001	9.2E-13				
**64**	rs820503	9	6667928	C/A	0.863	0.011	0.002	2.9E-11				
**65**	rs35234337	9	35661243	C/T	0.743	0.007	0.001	4.5E-08				
**66**	rs10868080	9	86626769	T/A	0.256	0.021	0.001	9.5E-63	rs296893	T/C	1	0.2783
**67**	rs56237852	9	100343212	C/A	0.826	0.008	0.002	1.0E-08				
**68**	rs62580766	9	113034490	T/C	0.182	0.010	0.002	1.6E-11				
**69**	rs79717793	10	5262267	G/A	0.845	0.023	0.002	4.8E-58				
**70**	rs3781085	10	13370958	T/G	0.585	0.006	0.001	3.6E-09				
**71**	rs3006593	10	31171626	C/G	0.380	0.007	0.001	2.7E-09	rs3006594	C/T	0.9874	0.3777
**72**	rs34390319	10	63960611	C/T	0.100	0.015	0.002	2.2E-13				
**73**	rs10822153	10	65056813	A/C	0.472	0.063	0.001	3.9E-714				
**74**	rs2259305	10	93615903	G/A	0.523	0.012	0.001	8.3E-28				
**75**	rs856534	10	94810665	A/G	0.387	0.011	0.001	1.3E-22				
**76**	rs7096937	10	113950418	T/C	0.269	0.009	0.001	1.4E-12	rs2255141	A/G	0.9585	0.3091
**77**	rs1037169	11	13361005	T/C	0.313	0.010	0.001	4.0E-16				
**78**	rs12797706	11	65561369	A/G	0.235	0.013	0.001	1.4E-22				
**79**	rs631695	11	69283303	T/G	0.418	0.018	0.001	2.0E-61				
**80**	rs10895277	11	102084940	A/G	0.659	0.010	0.001	1.3E-17				
**81**	rs2156805	11	122610568	G/A	0.508	0.006	0.001	4.4E-08				

**82**	rs1871395	12	21352315	A/G	0.848	0.032	0.002	2.7E-100				
**83**	rs75130744	12	25410741	G/C	0.928	0.029	0.002	3.0E-44	rs78444263	C/T	1	0.9274
**84**	rs12818938	12	53783182	T/G	0.832	0.008	0.002	1.1E-08	rs11170525	G/T	0.9781	0.838
**85**	rs540730	12	57807114	T/C	0.246	0.018	0.001	1.9E-48	rs1106766	T/C	0.9225	0.1938
**86**	rs11111274	12	102838128	G/A	0.263	0.008	0.001	1.3E-10	rs5742653	C/T	0.9735	0.2425
**87**	rs9738226	12	121423659	G/A	0.623	0.019	0.001	7.8E-61				
**88**	rs11621792	14	24871926	C/T	0.546	0.013	0.001	2.0E-28	rs11626929	C/T	0.8147	0.5189
**89**	rs2239222	14	73011885	G/A	0.350	0.012	0.001	8.8E-20				
**90**	rs13379043	14	74250126	C/T	0.280	0.008	0.001	2.9E-11				
**91**	rs28929474	14	94844947	T/C	0.020	0.133	0.004	1.2E-252				
**92**	rs3742366	14	104198351	C/T	0.346	0.009	0.001	3.6E-17				
**93**	rs7175361	15	41048058	A/G	0.158	0.009	0.002	3.4E-09				
**94**	rs139974673	15	44027885	T/C	0.975	0.078	0.004	4.6E-110				
**95**	rs56187480	15	63789479	G/A	0.655	0.014	0.001	1.2E-33				
**96**	rs8038465	15	73978337	T/C	0.425	0.007	0.001	4.6E-10				
**97**	rs72753908	15	83334856	C/T	0.925	0.013	0.002	1.9E-09				
**98**	rs11856926	15	96223649	G/A	0.551	0.011	0.001	1.1E-24				
**99**	rs56332871	15	96714816	A/C	0.272	0.031	0.001	6.0E-140	rs8023580	C/T	0.9626	0.3022
**100**	rs12928099	16	15150505	A/C	0.295	0.009	0.001	7.2E-13				
**101**	rs2288004	16	31054040	G/C	0.619	0.009	0.001	3.4E-16	rs9929899	C/A	1	0.6004
**102**	rs246192	16	58544295	G/C	0.480	0.008	0.001	9.8E-14	rs9783796	A/G	0.8455	0.4374
**103**	rs28650012	16	80497341	G/C	0.271	0.008	0.001	4.5E-11	rs4581712	A/C	0.9901	0.2763
**104**	rs8066941	17	9588450	T/G	0.761	0.016	0.001	7.7E-33				
**105**	rs2905801	17	29524974	T/C	0.705	0.013	0.001	1.2E-28	rs59015798	G/A	1	0.7326
**106**	rs11655704	17	47448172	C/T	0.315	0.030	0.001	1.5E-147				
**107**	rs7210574	17	73824121	C/T	0.329	0.010	0.001	2.3E-20				
**108**	rs36013981	17	79493307	A/G	0.604	0.006	0.001	8.2E-11	rs7342974	T/C	0.8738	0.5825
**109**	rs10871794	18	59342210	A/G	0.697	0.007	0.001	6.0E-09				

**110**	rs1788641	18	71949629	A/G	0.688	0.008	0.001	8.0E-12				
**111**	rs1640267	19	2789337	C/T	0.286	0.017	0.001	1.9E-45	rs941410	A/G	0.8807	0.3171
**112**	rs45512696	19	35550878	T/C	0.174	0.021	0.002	9.8E-47				
**113**	rs34255979	19	46384830	T/C	0.119	0.028	0.002	2.7E-63				
**114**	rs111981233	19	50016479	G/T	0.079	0.025	0.002	7.7E-36				
**115**	rs13042148	20	32298286	C/T	0.844	0.013	0.002	5.6E-20				
**116**	rs3795128	20	39774163	C/T	0.484	0.010	0.001	1.7E-23				
**117**	rs3746575	20	43058096	G/C	0.597	0.017	0.001	4.7E-49	rs3212198	T/C	0.8066	0.5606
**118**	rs6005840	22	29101357	A/G	0.327	0.016	0.001	1.5E-40				
**119**	rs738409	22	44324727	G/C	0.216	0.029	0.001	3.4E-98	rs2294915	T/C	0.8526	0.2525

SNP, single nucleotide polymorphism; Chr, chromosome; EA, effect allele; OA, other allele; EAF, effect allele frequency; SE, standard error.

Table S4. SNPs and proxies used for SHBG in women

NO.	SNP	CHR	POSITION (B37)	EA/OA	EAF	BETA	SE	P-VALUE	PROXY SNP	PROXY EA/OA	R^2	PROXY EAF
**1**	rs198358	1	11904076	C/T	0.248	0.008	0.001	9.0E-11				
**2**	rs74090351	1	61705898	A/G	0.069	0.019	0.002	5.9E-18	rs17311684	G/A	1	0.002
**3**	rs469721	1	91530001	C/T	0.803	0.011	0.002	5.4E-12				
**4**	rs1730862	1	107614003	G/A	0.342	0.023	0.001	1.7E-73	rs3108680	C/T	0.9337	0.331
**5**	rs267733	1	150958836	A/G	0.839	0.014	0.002	3.7E-21				
**6**	rs9426829	1	154592201	C/T	0.481	0.014	0.001	1.5E-30				
**7**	rs2064074	1	171061990	A/G	0.528	0.006	0.001	3.6E-08				
**8**	rs12138803	1	172348823	C/T	0.731	0.009	0.001	9.8E-10				
**9**	rs34331968	1	196659753	T/C	0.534	0.011	0.001	7.8E-20				
**10**	rs1418652	1	205646458	C/T	0.386	0.007	0.001	3.3E-08				
**11**	rs1223796	1	214323347	G/C	0.164	0.014	0.002	8.4E-19	rs1223801	G/A	0.9854	0.16
**12**	rs3001032	1	219727779	C/T	0.320	0.015	0.001	3.6E-29				
**13**	rs61830291	1	221001142	C/A	0.097	0.012	0.002	1.7E-08				
**14**	rs1870927	1	226426337	A/T	0.622	0.008	0.001	2.0E-11	rs2615799	T/C	0.8341	0.6789
**15**	rs1260326	2	27730940	C/T	0.607	0.035	0.001	1.7E-188				
**16**	rs11690748	2	48584575	C/G	0.623	0.008	0.001	9.7E-11	rs58660534	G/T	0.8995	0.6352
**17**	rs921153	2	61563408	A/G	0.159	0.009	0.002	2.8E-10				
**18**	rs13394092	2	85815954	C/T	0.170	0.008	0.002	1.4E-08				
**19**	rs13018007	2	114971913	G/A	0.082	0.013	0.002	1.2E-08	rs71416580	T/C	0.8532	0.0855
**20**	rs1128249	2	165528624	T/G	0.392	0.022	0.001	1.3E-73				
**21**	rs2364717	2	178101235	T/C	0.538	0.008	0.001	2.3E-12				
**22**	rs1047891	2	211540507	A/C	0.315	0.018	0.001	1.5E-44				
**23**	rs10189479	2	219287276	A/C	0.434	0.009	0.001	4.8E-14	rs2303561	T/C	0.8961	0.4066
**24**	rs2176040	2	227092802	A/G	0.354	0.015	0.001	2.7E-36				
**25**	rs1801282	3	12393125	G/C	0.120	0.024	0.002	8.9E-43	rs71304101	A/G	1	0.1203

**26**	rs10461018	3	46995242	T/C	0.420	0.011	0.001	2.5E-19	rs17476234	A/G	0.8499	0.3986
**27**	rs11130982	3	64728312	T/G	0.293	0.009	0.001	3.8E-11	rs9860221	G/A	0.9445	0.3002
**28**	rs4530527	3	86800085	C/A	0.361	0.008	0.001	1.8E-09				
**29**	rs17202341	3	105452593	G/A	0.348	0.007	0.001	1.3E-09				
**30**	rs11720108	3	123069058	T/C	0.249	0.008	0.001	5.4E-11				
**31**	rs687339	3	135932359	C/T	0.227	0.031	0.001	5.3E-104	rs6779146	C/T	0.9454	0.2356
**32**	rs9872754	3	138117985	C/T	0.840	0.010	0.002	9.0E-11				
**33**	rs9834503	3	149994882	A/C	0.540	0.008	0.001	1.9E-09	rs7433051	A/G	0.9412	0.5239
**34**	rs1126161	3	172228827	G/A	0.673	0.008	0.001	5.6E-09	rs3819773	G/A	1	0.6909
**35**	rs57158761	3	185371172	A/G	0.564	0.010	0.001	9.3E-18				
**36**	rs34311866	4	951947	T/C	0.824	0.011	0.002	6.1E-14				
**37**	rs925098	4	17919811	G/A	0.265	0.009	0.001	2.8E-09	rs724577	A/C	0.9763	0.2913
**38**	rs2970871	4	23890582	T/C	0.441	0.007	0.001	3.2E-08				
**39**	rs6531735	4	39686332	G/A	0.492	0.006	0.001	1.3E-08				
**40**	rs28636815	4	77197397	G/A	0.377	0.012	0.001	1.5E-21				
**41**	rs13150068	4	88203828	A/G	0.564	0.017	0.001	6.2E-47				
**42**	rs6831257	4	100018260	G/A	0.340	0.008	0.001	3.3E-11	rs2851270	G/A	0.9911	0.33
**43**	rs1433210	4	124766956	C/A	0.245	0.010	0.001	2.9E-12				
**44**	rs10857228	4	148979700	T/C	0.257	0.010	0.001	2.6E-13	rs12650033	G/A	0.9222	0.2664
**45**	rs28712547	4	157646955	G/A	0.321	0.010	0.001	2.9E-14				
**46**	rs11738093	5	53301425	A/G	0.748	0.014	0.001	6.0E-24	rs11741082	G/A	1	0.7167
**47**	rs40270	5	55804552	A/C	0.227	0.018	0.001	8.7E-35				
**48**	rs34651	5	72144005	T/C	0.918	0.011	0.002	3.0E-08	rs35585881	G/A	0.8109	0.9334
**49**	rs6860245	5	127367998	C/G	0.247	0.011	0.001	9.6E-14	rs2617617	C/T	0.9234	0.2058
**50**	rs2057655	5	131807624	A/G	0.187	0.011	0.002	2.4E-11				
**51**	rs1650527	5	158022724	C/T	0.768	0.014	0.001	4.1E-22				
**52**	rs6879874	5	176730775	T/A	0.724	0.008	0.001	1.8E-09	rs6885410	A/C	0.8549	0.7256
**53**	rs9366291	6	19381870	C/G	0.577	0.006	0.001	4.2E-08	rs10946313	T/C	0.8598	0.6262

**54**	rs28360642	6	41667506	A/C	0.839	0.020	0.002	5.8E-36				
**55**	rs150115323	6	117506408	G/C	0.372	0.006	0.001	4.1E-09	rs9372475	T/C	1	0.3569
**56**	rs58321169	6	126868567	C/T	0.732	0.010	0.001	5.6E-14	rs4273712	A/G	0.9658	0.7197
**57**	rs199607859	6	139835418	T/G	0.594	0.010	0.001	3.7E-17	rs17585887	C/T	0.9839	0.5726
**58**	rs1738386	6	151990235	C/T	0.380	0.008	0.001	1.9E-11	rs851972	A/G	1	0.4026
**59**	rs555754	6	160769423	A/G	0.468	0.018	0.001	1.7E-59				
**60**	rs2246223	7	6701189	T/C	0.555	0.008	0.001	3.6E-10	rs2243620	G/A	0.8261	0.5696
**61**	rs28459049	7	21567331	C/T	0.787	0.009	0.002	1.3E-10				
**62**	rs4563785	7	26349213	G/T	0.913	0.014	0.002	6.6E-12				
**63**	rs13237750	7	46456878	C/T	0.952	0.016	0.003	1.6E-09				
**64**	rs17492269	7	70047405	G/A	0.824	0.010	0.002	2.8E-09				
**65**	rs848476	7	77541673	G/A	0.292	0.010	0.001	1.1E-16				
**66**	rs10238028	7	99208899	G/A	0.067	0.014	0.002	6.5E-09				
**67**	rs6706	7	100471044	T/C	0.184	0.017	0.002	2.5E-29				
**68**	rs114949263	7	150498245	C/T	0.111	0.014	0.002	2.7E-14				
**69**	rs9644032	8	23414822	T/G	0.368	0.008	0.001	1.2E-11				
**70**	rs12543287	8	42334511	C/G	0.372	0.011	0.001	1.8E-18	rs10808961	A/G	0.8247	0.337
**71**	rs10095930	8	116974302	C/T	0.417	0.010	0.001	1.1E-15	rs7828742	A/G	0.8684	0.3986
**72**	rs4871015	8	128314516	A/G	0.581	0.008	0.001	5.1E-10				
**73**	rs820504	9	6668278	G/A	0.864	0.014	0.002	2.2E-16				
**74**	rs10961205	9	13722479	A/G	0.583	0.007	0.001	4.6E-08				
**75**	rs696825	9	86583076	T/C	0.252	0.024	0.001	4.5E-63				
**76**	rs62580766	9	113034490	T/C	0.181	0.011	0.002	7.5E-16				
**77**	rs4837794	9	123507855	T/C	0.332	0.010	0.001	1.4E-16	rs7036196	A/G	0.8045	0.3012
**78**	rs8176741	9	136131461	G/A	0.938	0.016	0.003	1.7E-10	rs7470777	A/G	0.873	0.9215
**79**	rs7475279	10	5252866	A/C	0.846	0.018	0.002	1.3E-30				
**80**	rs899865	10	36473044	T/C	0.600	0.007	0.001	1.0E-08	rs10827616	C/T	0.9198	0.6153
**81**	rs1530439	10	63645959	T/G	0.309	0.011	0.001	9.7E-19				

**82**	rs2068888	10	94839642	A/G	0.451	0.013	0.001	2.0E-26				
**83**	rs10883451	10	101924418	C/T	0.499	0.009	0.001	8.2E-11				
**84**	rs140312320	10	103992418	G/A	0.933	0.017	0.002	1.1E-11				
**85**	rs35198068	10	114754784	T/C	0.710	0.011	0.001	2.0E-19				
**86**	rs1037169	11	13361005	T/C	0.313	0.013	0.001	1.2E-24				
**87**	rs174537	11	61552680	G/T	0.654	0.012	0.001	5.5E-21				
**88**	rs12804411	11	69284200	T/C	0.231	0.014	0.001	2.6E-24				
**89**	rs11021232	11	95320808	T/C	0.820	0.013	0.002	4.0E-17	rs4409785	T/C	0.9504	0.8101
**90**	rs10893876	11	128353007	C/T	0.766	0.008	0.001	3.5E-08				
**91**	rs17887160	12	6877721	C/T	0.720	0.009	0.001	7.4E-12	rs11064373	G/A	0.8188	0.7773
**92**	rs4149056	12	21331549	T/C	0.849	0.030	0.002	1.5E-74				
**93**	rs11047237	12	24206326	A/T	0.965	0.028	0.003	8.2E-17	rs36124182	A/G	0.8991	0.9483
**94**	rs4307773	12	51144432	T/C	0.419	0.014	0.001	5.9E-31				
**95**	rs8756	12	66359752	C/A	0.485	0.008	0.001	1.7E-13				
**96**	rs3751129	12	102455729	A/G	0.216	0.011	0.001	1.7E-13	rs2373567	C/T	1	0.2157
**97**	rs7139079	12	121415293	A/G	0.593	0.013	0.001	5.4E-28				
**98**	rs12311848	12	124486851	G/A	0.333	0.014	0.001	1.6E-29				
**99**	rs9556403	13	95236825	G/A	0.350	0.007	0.001	6.8E-10				
**100**	rs7321688	13	115000365	C/A	0.767	0.009	0.001	1.7E-09				
**101**	rs11621792	14	24871926	C/T	0.548	0.026	0.001	4.0E-102	rs11626929	C/T	0.8147	0.5189
**102**	rs2239222	14	73011885	G/A	0.349	0.010	0.001	2.1E-16				
**103**	rs13379043	14	74250126	C/T	0.278	0.011	0.001	2.2E-16				
**104**	rs28929474	14	94844947	T/C	0.020	0.061	0.004	3.1E-43				
**105**	rs2498786	14	105262368	C/G	0.385	0.011	0.001	5.5E-19	rs4983559	G/A	0.8284	0.3917
**106**	rs275177	15	39449003	C/T	0.150	0.010	0.002	5.5E-09				
**107**	rs139974673	15	44027885	T/C	0.974	0.054	0.004	1.4E-49				
**108**	rs12438742	15	61947280	G/C	0.570	0.007	0.001	2.9E-08	rs2443058	C/A	0.9519	0.5905
**109**	rs12906447	15	96224270	C/T	0.552	0.009	0.001	4.3E-14				

**110**	rs56332871	15	96714816	A/C	0.272	0.039	0.001	9.2E-188	rs8023580	C/T	0.9626	0.3022
**111**	rs4122352	16	15174571	A/G	0.296	0.011	0.001	1.8E-16	rs11644601	C/T	0.9712	0.2903
**112**	rs858519	17	7531965	C/T	0.557	0.099	0.001	1.7E-1533				
**113**	rs2525570	17	29681245	G/A	0.601	0.007	0.001	1.0E-09				
**114**	rs140302625	17	47379867	T/G	0.087	0.062	0.002	4.2E-193				
**115**	rs72844546	17	73149850	C/T	0.346	0.010	0.001	3.1E-18	rs7218706	T/C	0.8236	0.339
**116**	rs10153315	17	79481772	T/C	0.581	0.009	0.001	7.5E-16	rs7342974	T/C	0.9758	0.5825
**117**	rs4327143	18	71925113	A/G	0.714	0.009	0.001	1.7E-11	rs1790813	T/G	0.93	0.7336
**118**	rs7250869	19	33887405	C/T	0.690	0.011	0.001	2.7E-20	rs889140	A/G	0.8385	0.6531
**119**	rs2018519	19	35559787	C/T	0.181	0.020	0.002	3.4E-40				
**120**	rs73036519	19	45748362	G/C	0.700	0.009	0.001	7.3E-10	rs151165225	C/T	0.8285	0.7396
**121**	rs34255979	19	46384830	T/C	0.121	0.028	0.002	7.9E-52				
**122**	rs59774409	19	50016748	T/C	0.082	0.018	0.002	5.2E-16				
**123**	rs1741344	20	4101800	T/C	0.634	0.007	0.001	3.4E-09				
**124**	rs13042148	20	32298286	C/T	0.845	0.016	0.002	1.9E-21				
**125**	rs6073431	20	43040569	T/C	0.532	0.017	0.001	6.2E-43				
**126**	rs4810580	20	45594295	T/G	0.782	0.010	0.002	6.2E-10				
**127**	rs16995626	20	49540925	C/T	0.072	0.018	0.002	3.7E-15				
**128**	rs5753111	22	30779211	T/C	0.291	0.013	0.001	4.4E-23				
**129**	rs3747207	22	44324855	A/G	0.214	0.017	0.002	1.0E-30	rs2294915	T/C	0.838	0.2525

SNP, single nucleotide polymorphism; Chr, chromosome; EA, effect allele; OA, other allele; EAF, effect allele frequency; SE, standard error.

Table S5. SNPs and proxies used for bioavailable testosterone in men

NO.	SNP	CHR	POSITION (B37)	EA/OA	EAF	BETA	SE	P-VALUE	PROXY SNP	PROXY EA/OA	R^2	PROXY EAF
**1**	rs71519251	1	163251833	A/G	0.167	0.024	0.004	1.5E-09	rs12724399	C/T	0.9277	0.1829
**2**	rs6729954	2	18286651	T/A	0.435	0.019	0.003	3.3E-10	rs6742679	A/G	0.9837	0.4155
**3**	rs829593	2	30641234	G/A	0.687	0.018	0.003	1.5E-08				
**4**	rs6718154	2	180497923	T/C	0.275	0.032	0.003	1.2E-22				
**5**	rs2011425	2	234627608	T/G	0.920	0.050	0.006	6.4E-20				
**6**	rs1112195	3	24085166	G/A	0.495	0.018	0.003	4.7E-10				
**7**	rs9824196	3	28807441	T/G	0.721	0.026	0.003	4.1E-16				
**8**	rs3821866	3	53805577	G/C	0.622	0.022	0.003	4.1E-14	rs11918583	A/G	0.9956	0.662
**9**	rs13065463	3	61662996	G/A	0.869	0.032	0.005	8.8E-13				
**10**	rs10510939	3	65507808	C/T	0.375	0.017	0.003	1.6E-08				
**11**	rs34040779	3	107235109	T/C	0.924	0.035	0.006	2.2E-09	rs16853512	A/G	1	0.9215
**12**	rs4678408	3	138053187	G/A	0.630	0.026	0.003	7.6E-17	rs1002766	A/G	0.9077	0.666
**13**	rs7679843	4	22028079	G/C	0.095	0.050	0.005	3.8E-22	rs11728819	A/C	0.8863	0.1064
**14**	rs4274916	4	69988378	C/T	0.542	0.017	0.003	3.2E-09				
**15**	rs950716	5	135680540	A/G	0.863	0.034	0.004	3.1E-14				
**16**	rs2961853	5	165932048	C/T	0.469	0.019	0.003	3.2E-10				
**17**	rs34192788	6	17416258	T/A	0.690	0.020	0.003	4.2E-09	rs17379883	G/A	1	0.672
**18**	rs7454964	6	52728059	T/C	0.428	0.017	0.003	1.8E-08	rs11969435	T/C	0.9959	0.4254
**19**	rs9322822	6	105369598	C/T	0.679	0.049	0.003	2.1E-52				
**20**	rs2184968	6	126760994	C/T	0.451	0.020	0.003	9.1E-11				
**21**	rs10279715	7	40870935	A/G	0.538	0.022	0.003	3.1E-13				
**22**	rs55795858	7	146123500	C/T	0.329	0.018	0.003	1.5E-08	rs2888335	C/T	0.8808	0.2753
**23**	rs2631864	8	21112084	G/A	0.104	0.033	0.005	8.3E-12				
**24**	rs4872310	8	25247181	G/A	0.753	0.023	0.004	3.7E-12				
**25**	rs4562360	8	61704817	G/A	0.757	0.032	0.004	3.6E-20				

**26**	rs71529289	8	77879487	C/T	0.748	0.036	0.003	2.0E-25				
**27**	rs4483209	9	1960629	T/G	0.472	0.017	0.003	4.3E-09	rs7857865	A/G	0.9562	0.4374
**28**	rs745486	9	11242155	C/T	0.719	0.021	0.003	1.2E-10				
**29**	rs10738700	9	24973797	A/G	0.569	0.020	0.003	5.6E-11				
**30**	rs912202	9	77225603	C/G	0.343	0.040	0.003	2.5E-38	rs62569142	A/G	1	0.3817
**31**	rs2090409	9	108967088	C/A	0.684	0.031	0.003	1.7E-21				
**32**	rs7872329	9	131956152	A/T	0.689	0.017	0.003	3.8E-08	rs6478869	C/T	1	0.7147
**33**	rs7912521	10	67262089	C/T	0.416	0.061	0.003	6.2E-94	rs7097461	A/G	0.9798	0.4284
**34**	rs4919686	10	104592249	A/C	0.710	0.023	0.003	3.9E-13				
**35**	rs7915430	10	121660465	T/G	0.798	0.021	0.004	9.7E-09				
**36**	rs2035837	11	29200527	T/C	0.852	0.073	0.004	6.7E-67				
**37**	rs55765314	11	72360935	C/A	0.838	0.025	0.004	4.6E-10				
**38**	rs503542	11	118590743	G/A	0.450	0.018	0.003	5.8E-10				
**39**	rs10892924	11	122773715	T/A	0.568	0.039	0.003	2.8E-37	rs4936759	C/T	0.9897	0.5696
**40**	rs61932784	12	114132310	C/A	0.783	0.022	0.004	4.0E-09				
**41**	rs10137488	14	35797122	C/T	0.027	0.053	0.010	2.1E-08				
**42**	rs1272131	14	60886150	C/T	0.387	0.027	0.003	1.4E-17	rs10483727	T/C	0.9713	0.4036
**43**	rs1454836	15	47551054	T/A	0.601	0.017	0.003	3.4E-08	rs766132	C/T	0.9107	0.6083
**44**	rs17703883	15	51530097	C/T	0.255	0.044	0.004	7.5E-40				
**45**	rs13835	15	89056040	A/C	0.424	0.019	0.003	4.9E-10				
**46**	rs62041532	16	73922719	G/T	0.213	0.023	0.004	1.6E-10				
**47**	rs1799941	17	7533423	G/A	0.739	0.034	0.003	7.7E-23	rs149932962	G/A	0.8877	0.7604
**48**	rs2668776	18	44750365	C/T	0.469	0.029	0.003	1.7E-22				
**49**	rs2327121	20	8878250	C/G	0.657	0.018	0.003	1.3E-08	rs6056230	G/T	0.9906	0.6998
**50**	rs7265992	20	33525407	G/A	0.821	0.032	0.004	4.0E-16				
**51**	rs11703376	22	49678713	T/C	0.270	0.040	0.003	5.9E-32	rs6009583	T/C	0.9788	0.2465

SNP, single nucleotide polymorphism; Chr, chromosome; EA, effect allele; OA, other allele; EAF, effect allele frequency; SE, standard error.

Table S6. SNPs and proxies used for bioavailable testosterone in women

NO.	SNP	CHR	POSITION (B37)	EA/OA	EAF	BETA	SE	P-VALUE	PROXY SNP	PROXY EA/OA	R^2	PROXY EAF
**1**	rs1989147	1	7909373	C/T	0.807	0.024	0.003	7.0E-14				
**2**	rs6684361	1	101737743	C/T	0.307	0.040	0.003	5.4E-48				
**3**	rs12564492	1	168234645	A/G	0.691	0.015	0.003	5.0E-09	rs4656150	C/T	0.9678	0.6909
**4**	rs2266782	1	171076966	A/G	0.417	0.016	0.003	2.6E-11				
**5**	rs2152318	1	179293511	T/C	0.245	0.032	0.003	1.8E-27				
**6**	rs34269793	2	12555164	C/T	0.053	0.033	0.006	2.2E-10				
**7**	rs62144584	2	25004937	T/C	0.728	0.019	0.003	1.6E-10				
**8**	rs2374456	2	43271621	G/C	0.546	0.016	0.003	7.4E-10	rs11685806	T/C	0.8441	0.5457
**9**	rs13030651	2	43508205	G/A	0.449	0.017	0.003	7.0E-12				
**10**	rs13430258	2	62535082	G/T	0.534	0.016	0.003	1.9E-10				
**11**	rs1515098	2	227073854	T/C	0.680	0.015	0.003	9.0E-09				
**12**	rs62231822	3	14423060	C/T	0.901	0.029	0.004	8.3E-12				
**13**	rs13072623	3	20163556	G/A	0.433	0.015	0.003	4.5E-09				
**14**	rs6788984	3	41107173	A/G	0.856	0.020	0.004	2.4E-08				
**15**	rs687339	3	135932359	T/C	0.764	0.040	0.003	4.1E-39	rs6779146	T/C	0.9454	0.7644
**16**	rs7633673	3	152084243	G/A	0.594	0.018	0.003	2.6E-13				
**17**	rs4368453	3	156852141	T/C	0.302	0.017	0.003	3.6E-09				
**18**	rs12645584	4	17919066	T/C	0.707	0.017	0.003	3.9E-09	rs2320299	A/G	0.9764	0.7068
**19**	rs71633359	4	88183820	C/T	0.258	0.023	0.003	4.0E-16	rs13130929	A/G	0.8941	0.2584
**20**	rs12189146	5	35239886	G/A	0.950	0.032	0.006	1.8E-08				
**21**	rs40270	5	55804552	C/A	0.773	0.019	0.003	4.0E-10				
**22**	rs1119208	5	76488613	C/T	0.649	0.017	0.003	1.2E-11				
**23**	rs168189	5	77974427	T/C	0.286	0.020	0.003	5.4E-13	rs784420	G/A	0.9821	0.2863
**24**	rs12658172	5	124205385	G/C	0.835	0.030	0.004	1.8E-18	rs60775983	A/G	1	0.835
**25**	rs1432679	5	158244083	C/T	0.446	0.013	0.003	4.7E-08				

**26**	rs62396733	6	41679691	T/C	0.137	0.021	0.004	5.7E-09				
**27**	rs1214759	6	43352980	G/A	0.679	0.018	0.003	2.8E-12				
**28**	rs2397112	6	52684333	A/G	0.576	0.016	0.003	4.0E-10	rs6932500	G/A	0.9959	0.5755
**29**	rs9399469	6	144318529	A/T	0.610	0.020	0.003	2.6E-14	rs9376802	T/C	0.9547	0.6103
**30**	rs4869893	6	157117322	C/A	0.282	0.016	0.003	1.1E-08				
**31**	rs45446698	7	99332948	T/G	0.951	0.161	0.006	5.1E-148	rs148982377	T/C	0.81	0.9513
**32**	rs10504255	8	59398461	G/A	0.337	0.017	0.003	6.1E-10				
**33**	rs113347955	8	97136852	G/A	0.976	0.045	0.008	1.2E-08				
**34**	rs35783704	8	105966258	A/G	0.101	0.033	0.004	4.7E-16				
**35**	rs12543598	8	143955318	T/G	0.407	0.020	0.003	6.6E-15	rs4581033	A/C	0.9918	0.4066
**36**	rs10757893	9	29599001	A/G	0.532	0.014	0.003	9.3E-09	rs10757895	A/G	0.8275	0.5318
**37**	rs1171617	10	61467182	T/G	0.767	0.030	0.003	3.6E-23				
**38**	rs7089122	10	93647412	T/C	0.199	0.024	0.003	1.7E-14	rs2259287	G/A	0.9204	0.1988
**39**	rs9527	10	104623578	C/T	0.758	0.023	0.003	5.9E-15				
**40**	rs7078330	10	122834163	T/C	0.088	0.031	0.004	5.7E-13				
**41**	rs6486122	11	13361524	T/C	0.690	0.017	0.003	1.7E-09				
**42**	rs11031005	11	30226356	C/T	0.144	0.023	0.004	1.5E-10				
**43**	rs113172275	11	62905115	C/T	0.066	0.049	0.005	1.4E-22				
**44**	rs3814707	11	65560785	G/A	0.758	0.020	0.003	5.5E-11				
**45**	rs850294	11	123437669	T/C	0.114	0.034	0.004	5.2E-18				
**46**	rs4149056	12	21331549	C/T	0.151	0.043	0.004	3.0E-35				
**47**	rs11047261	12	24257228	G/A	0.041	0.037	0.006	6.4E-09				
**48**	rs56205943	12	57679414	G/A	0.759	0.021	0.003	3.9E-13				
**49**	rs7301634	12	98901200	A/G	0.810	0.019	0.003	2.8E-09				
**50**	rs10778215	12	103537266	A/T	0.506	0.024	0.003	2.6E-22	rs4764939	T/C	0.9764	0.506
**51**	rs7139079	12	121415293	G/A	0.407	0.022	0.003	1.1E-16				
**52**	rs2954111	12	122134415	C/T	0.361	0.017	0.003	3.1E-10				
**53**	rs629042	13	22318506	C/G	0.603	0.028	0.003	7.4E-29	rs631660	G/T	0.9959	0.6034

**54**	rs17245822	13	73131694	C/A	0.360	0.014	0.003	3.6E-08	rs1337985	C/T	0.9957	0.3598
**55**	rs11621792	14	24871926	T/C	0.481	0.025	0.003	1.4E-22	rs11626929	T/C	0.8147	0.4811
**56**	rs112635299	14	94838142	G/T	0.979	0.102	0.009	1.1E-31				
**57**	rs7183977	15	40377092	C/T	0.324	0.029	0.003	7.8E-27	rs4924416	T/G	0.9462	0.3241
**58**	rs10851395	15	40718534	C/T	0.524	0.019	0.003	1.1E-13				
**59**	rs62025141	15	79850183	A/G	0.855	0.026	0.004	1.2E-13				
**60**	rs56332871	15	96714816	C/A	0.698	0.030	0.003	9.7E-27	rs8023580	T/C	0.9626	0.6978
**61**	rs388430	16	4135562	C/T	0.688	0.017	0.003	2.6E-10	rs387212	T/C	0.9954	0.6879
**62**	rs8046391	16	30836648	C/G	0.305	0.017	0.003	8.8E-10	rs28421305	A/G	1	0.3052
**63**	rs58072681	16	81590541	C/T	0.070	0.061	0.005	8.3E-36				
**64**	rs727428	17	7537792	T/C	0.444	0.095	0.003	8.3E-309				
**65**	rs11653686	17	47362991	C/T	0.913	0.066	0.004	8.3E-48				
**66**	rs7239564	18	71967031	C/T	0.856	0.031	0.004	5.0E-18				
**67**	rs1640272	19	2800192	A/T	0.683	0.021	0.003	3.7E-14	rs941410	G/A	0.9021	0.6829
**68**	rs7248104	19	7224431	G/A	0.584	0.016	0.003	4.5E-10				
**69**	rs34255979	19	46384830	C/T	0.879	0.023	0.004	1.5E-09				
**70**	rs6020423	20	48909667	C/T	0.760	0.025	0.003	2.3E-18				
**71**	rs6008259	22	46633782	G/A	0.821	0.021	0.003	5.3E-11				

SNP, single nucleotide polymorphism; Chr, chromosome; EA, effect allele; OA, other allele; EAF, effect allele frequency; SE, standard error.

TABLE S7. Study characteristics and SNP effects for estradiol

STUDY	SEX	N	SNP	GENE	EA/OA	EAF	BETA*	SE	P-VALUE	ADJUSTMENTS	ANCESTRY
**Thompson et al, (2016)**	Females	2,767	rs727479	CYP19A1	A/C	0.656	0.096	0.018	7.40E-8	Laboratory batch, study, age at blood draw, BMI, HRT use and menopausal status (2–5 years, or >5 years since menopause)	European
**Eriksson et al, (2018)**	Males	11,097	rs727479	CYP19A1	A/C	0.63	1.39	0.12	8.2E-30	Age, BMI	European

*Beta for the study by Thompson is in log-transformed estradiol concentration, for Eriksson et al it is the effect in picogram per milliliter. SNP, single nucleotide polymorphism; EA, Effect allele; OA, Other allele; EAF, Effect allele frequency; SE, Standard error; BMI, Body mass index.

Table S8. Mendelian randomization results after cluster filtering

EXPOSURE	SEX	UNIT	METHOD	SNPS	ODDS RADIO	95% CI	P-VALUE	Q	P-HET
**Bioavailable testosterone**	F	1 s.d. increase	IVW (random effects)	17	1.24	0.81-1.91	0.313	12.33	0.721
			Weighted median	17	1.38	0.72-2.66	0.337		
			MR-Egger intercept	17	β= -0.00113	s.e.= 0.01715	0.948		
			MR-Egger	17	1.27	0.58-2.81	0.561		
**SHBG**	F	1 s.d. increase	IVW (random effects)	126	1.21	1.05 -1.40	0.008	112.20	0.787
			Weighted median	126	1.31	1.02-1.70	0.032		
			MR-Egger intercept	126	β=-0.0074636	s.e.=0.0075638	0.325		
			MR-Egger	126	1.27	1.00-1.61	0.058		

SNPs used for bioavailable testosterone have effects independent of SHBG. SNPs used for SHBG have a primary effect on SHBG, and secondary (opposite) effects on bioavailable testosterone. 'P-het' represents the p-value belonging to the Q-statistic. F, Females; M, Males; s.e., standard error; s.d., standard deviation; N/A, Not Applicable;

Table S9. Multivariable Mendelian randomization results

EXPOSURE	SEX	UNIT	METHOD	SNPS	ODDS RADIO	95% CI	P-VALUE	Q	P-HET	P-EGGER
**Bioavailable testosterone**	F	1 s.d. increase	MV-IVW (random effects)	162	1.02	0.61-1.69	0.952	138.24	0.89	0.15
**SHBG**	F	1 s.d. increase	MV-IVW (random effects)	162	1.17	0.94 -1.46	0.169			

Estimates represent the estimated direct causal effect of the exposure, while accounting for the other exposure. 'P-het' represents the p-value belonging to the Q-statistic. 'P-Egger' represents the p-value belonging to the Egger-intercept. F, Females; M, Males; s.e., standard error; s.d., standard deviation; N/A, Not Applicable.

Table S10. Mendelian randomization results for the different exposures on aSAH risk among women

EXPOSURE	SEX	UNIT	METHOD	SNPS	ODDS RADIO	95% CI	P-VALUE	Q	P-HET
**Age at menarche**	F	1-year increase	IVW (random effects)	163	0.96	0.82-1.12	0.576	159.78	0.534
			Weighted median	163	1.04	0.82-1.33	0.737		
			MR-Egger intercept	163	β=-0.0074636	s.e.=0.0075638	0.325		
			MR-Egger	163	1.15	0.78-1.69	0.493		
			MR-PRESSO	N/A	N/A	N/A	N/A		
**Age at menopause**	F	1-year increase	IVW (random effects)	112	1.01	0.96-1.06	0.807	97.26	0.821
			Weighted median	112	1.00	0.92-1.09	0.975		
			MR-Egger intercept	112	β=0.008318	s.e.=0.007939	0.297		
			MR-Egger	112	0.95	0.85-1.07	0.415		
			MR-PRESSO	N/A	N/A	N/A	N/A		
**Bioavailable testosterone**	F	1 s.d. increase	IVW (random effects)	71	0.73	0.55-0.95	0.020	67.92	0.548
			Weighted median	71	0.64	0.42-0.98	0.038		
			MR-Egger intercept	71	β=-0.00023	s.e.=0.008943	0.979		
			MR-Egger	71	0.73	0.44-1.21	0.229		
			MR-PRESSO	N/A	N/A	N/A	N/A		
**SHBG**	F	1 s.d. increase	IVW (random effects)	129	1.18	1.05-1.34	0.007	112.44	0.835

			Weighted median	129	1.27	1.02-1.59	0.035		
			MR-Egger intercept	129	β=-0.00257	s.e.=0.005459	0.639		
			MR-Egger	129	1.23	1.00-1.52	0.053		
			MR-PRESSO	N/A	N/A	N/A	N/A		
**SHBG (unadjusted for BMI)**	F	1 s.d. increase	IVW (random effects)	129	1.24	1.05-1.47	0.009	112.91	0.827
			Weighted median	129	1.36	1.02-1.83	0.039		
			MR-Egger intercept	129	β=-0.00151	s.e.=0.0053	0.776		
			MR-Egger	129	1.28	0.98-1.68	0.077		
			MR-PRESSO	N/A	N/A	N/A	N/A		
**Estradiol**	F	1 s.d. increase	Wald estimate	1	0.85	0.43-1.67	0.642		

‘P-het’ represents the p-value belonging to the Q-statistic. F, Females; M, Males; s.e., standard error; s.d., standard deviation; N/A, Not Applicable; BMI, body mass index.

Table S11. Sensitivity analyses for the different exposures on aSAH risk among men

EXPOSURE	SEX	UNIT	METHOD	SNPS	ODDS RADIO	95% CI	P-VALUE	Q	P-HET
**Age at menarche**	M	1-year increase	IVW (random effects)	163	1.12	0.92-1.36	0.279	156.36	0.610
			Weighted median	163	1.23	0.87-1.73	0.244		
			MR-Egger intercept	163	β=-0.00035	s.e.=0.009781	0.971		
			MR-Egger	163	1.13	0.68-1.86	0.644		
			MR-PRESSO	N/A	N/A	N/A	N/A		
**Age at menopause**	M	1-year increase	IVW (random effects)	112	1.04	0.97-1.12	0.235	119.53	0.273
			Weighted median	112	1.05	0.93-1.18	0.451		
			MR-Egger intercept	112	β=0.002552	s.e.= 0.01072	0.812		
			MR-Egger	112	1.03	0.88-1.19	0.732		
			MR-PRESSO	N/A	N/A	N/A	N/A		
**Bioavailable testosterone**	M	1 s.d. increase	IVW (random effects)	51	0.86	0.56-1.34	0.516	58.65	0.188
			Weighted median	51	0.80	0.42-1.51	0.489		
			MR-Egger intercept	51	β=0.011498	s.e.=0.018964	0.547		
			MR-Egger	51	0.63	0.21-1.90	0.419		
			MR-PRESSO	N/A	N/A	N/A	N/A		
**SHBG**	M	1 s.d. increase	IVW (random effects)	119	0.97	0.80-1.16	0.703	135.04	0.135

			Weighted median	119	1.09	0.81-1.48	0.561
			MR-Egger intercept	119	β=-0.00984	s.e.=0.008511	0.250
			MR-Egger	119	1.12	0.82-1.54	0.563
			MR-PRESSO	N/A	N/A	N/A	N/A
**Estradiol**	M	1 s.d. increase	Wald estimate	1	1.02	0.96-1.08	0.589

'P-het' represents the p-value belonging to the Q-statistic. F, Females; M, Males; s.e., standard error; N/A, Not Applicable;

Table S12. Mendelian randomization results after MR-Steiger filtering

EXPOSURE	SEX	UNIT	METHOD	SNPS	ODDS RADIO	95% CI	P-VALUE	Q	P-HET
**Age at menarche**	F	1-year increase	IVW (random effects)	117	0.95	0.78-1.16	0.633	125.16	0.264
			Weighted median	117	0.87	0.64-1.17	0.353		
			MR-Egger intercept	117	β=-0.00156	s.e.= 0.01011	0.878		
			MR-Egger	117	0.99	0.57-1.71	0.975		
			MR-PRESSO	N/A	N/A	N/A	N/A		
**Age at menopause**	F	1-year increase	IVW (random effects)	96	1.00	0.95-1.05	0.948	79.09	0.880
			Weighted median	96	1.01	0.92-1.10	0.875		
			MR-Egger intercept	96	β=0.00470	s.e.= 0.00836	0.575		
			MR-Egger	96	0.97	0.87-1.09	0.603		
			MR-PRESSO	N/A	N/A	N/A	N/A		
**Bioavailable testosterone**	F	1 s.d. increase	IVW (random effects)	56	0.69	0.52-0.93	0.016	53.89	0.517
			Weighted median	56	0.65	0.41-1.03	0.068		
			MR-Egger intercept	56	β=-0.00625	s.e.= 0.00955	0.516		
			MR-Egger	56	0.80	0.48-1.35	0.411		
			MR-PRESSO	N/A	N/A	N/A	N/A		
**Bioavailable testosterone**	M	1 s.d. increase	IVW (random effects)	35	1.04	0.62-1.74	0.881	39.41	0.241

			Weighted median	35	0.81	0.38-1.68	0.568		
			MR-Egger intercept	35	β=0.00940	s.e.= 0.02497	0.709		
			MR-Egger	35	0.81	0.20-3.31	0.663		
			MR-PRESSO	N/A	N/A	N/A	N/A		
**SHBG**	F	1 s.d. increase	IVW (random effects)	112	1.19	1.05-1.34	0.007	93.06	0.891
			Weighted median	112	1.02	1.02-1.58	0.030		
			MR-Egger intercept	112	β=-0.00197	s.e.= 0.0058	0.735		
			MR-Egger	112	1.23	1.00-1.52	0.056		
			MR-PRESSO	N/A	N/A	N/A	N/A		
**SHBG**	M	1 s.d. increase	IVW (random effects)	92	0.89	0.73-1.10	0.296	107.40	0.115
			Weighted median	92	1.08	0.77-1.49	0.664		
			MR-Egger intercept	92	β= -0.01112	s.e.= 0.00998	0.268		
			MR-Egger	92	1.06	0.74-1.54	0.746		
			MR-PRESSO	N/A	N/A	N/A	N/A		

‘P-het’ represents the p-value belonging to the Q-statistic. F, Females; M, Males; s.e., standard error; s.d., standard deviation; N/A, Not Applicable;

International Stroke Genetics Consortium (ISGC) Intracranial Aneurysm Working Group:

Mark K. Bakker[1], Romain Bourcier[2,3], Robin G. Walters[4,5], Rainer Malik[6], Martin Dichgans[6,7,8], Muralidharan Sargurupremraj[9,10], Turgut Tatlisumak[11], Stéphanie Debette[9,10], Gabriel J.E. Rinkel[1], Bradford B. Worrall[12], Joanna Pera[13], Agnieszka Slowik[13], Joseph P. Broderick[14], David J. Werring[15], Daniel Woo[14], Philippe Bijlenga[16], Yoichiro Kamatani[17], Ynte M. Ruigrok[1]

[1]Department of Neurology and Neurosurgery, University Medical Center Utrecht Brain Center, Utrecht University, Utrecht, The Netherlands. [2]Université de Nantes, CHU Nantes, INSERM, CNRS, l'institut du thorax, Nantes, France. [3]CHU Nantes, Department of Neuroradiology, Nantes, France. [4]Clinical Trial Service Unit and Epidemiological Studies Unit, Nuffield Department of Population Health, University of Oxford, Oxford, U.K. [5]Medical Research Council Population Health Research Unit, University of Oxford, Oxford, U.K. [6]Institute for Stroke and Dementia Research, University Hospital, Ludwig-Maximilians-University, Munich. [7]Munich Cluster for Systems Neurology (SyNergy), Munich, Germany. [8]Deutsches Zentrum für Neurodegenerative Erkrankungen (DZNE), Munich, Germany. [9]INSERM U1219 Bordeaux Population Health Research Center, University of Bordeaux, Bordeaux, France. [10]Department of Neurology, Institute for Neurodegenerative Disease, Bordeaux University Hospital, Bordeaux, France. [11]Department of Clinical Neuroscience at Institute of Neuroscience and Physiology, University of Gothenburg, Sweden. [12]Departments of Neurology and Public Health Sciences, University of Virginia School of Medicine, Charlottesville, VA, USA. [13]Department of Neurology, Faculty of Medicine, Jagiellonian University Medical College, ul. Botaniczna 3, 31-503, Krakow, Poland. [14]University of Cincinnati College of Medicine, Cincinnati, OH, USA. [15]Stroke Research Centre, University College London Queen Square Institute of Neurology, London, UK. [16]Neurosurgery Division, Department of Clinical Neurosciences, Faculty of Medicine, Geneva University Hospitals, Geneva, Switzerland. [17]Graduate School of Frontier Sciences, The University of Tokyo, Tokyo, Japan.

STROBE-MR checklist

Item No.	Section		Checklist item	Section (paragraph number)
1	**TITLE and ABSTRACT**		Indicate Mendelian randomization as the study's design in the title and/or the abstract if that is a main purpose of the study	Title page
	**INTRODUCTION**			
2	**Background**		Explain the scientific background and rationale for the reported study. What is the exposure? Is a potential causal relationship between exposure and outcome plausible? Justify why MR is a helpful method to address the study question	Introduction (paragraphs 1-3)
3	**Objectives**		State specific objectives clearly, including pre-specified causal hypotheses (if any). State that MR is a method that, under specific assumptions, intends to estimate causal effects	Introduction (paragraph 3)
	**METHODS**			
4	**Study design and data sources**		Present key elements of the study design early in the article. Consider including a table listing sources of data for all phases of the study. For each data source contributing to the analysis, describe the following:	
		a)	Setting: Describe the study design and the underlying population, if possible. Describe the setting, locations, and relevant dates, including periods of recruitment, exposure, follow-up, and data collection, when available.	Methods (paragraph 2,3)
		b)	Participants: Give the eligibility criteria, and the sources and methods of selection of participants. Report the sample size, and whether any power or sample size calculations were carried out prior to the main analysis	Methods (paragraph 2,3), Table 1
		c)	Describe measurement, quality control and selection of genetic variants	Methods (paragraph 2,3)
		d)	For each exposure, outcome, and other relevant variables, describe methods of assessment and diagnostic criteria for diseases	Table 1
		e)	Provide details of ethics committee approval and participant informed consent, if relevant	N/A

5	**Assumptions**		Explicitly state the three core IV assumptions for the main analysis (relevance, independence and exclusion restriction) as well assumptions for any additional or sensitivity analysis	Methods (paragraph 4)
6	**Statistical methods: main analysis**		Describe statistical methods and statistics used	
		a)	Describe how quantitative variables were handled in the analyses (i.e., scale, units, model)	Table 1
		b)	Describe how genetic variants were handled in the analyses and, if applicable, how their weights were selected	Methods (paragraphs 4,5)
		c)	Describe the MR estimator (e.g. two-stage least squares, Wald ratio) and related statistics. Detail the included covariates and, in case of two-sample MR, whether the same covariate set was used for adjustment in the two samples	Methods (paragraphs 4,5)
		d)	Explain how missing data were addressed	N/A
		e)	If applicable, indicate how multiple testing was addressed	Methods (paragraph 6)
7	**Assessment of assumptions**		Describe any methods or prior knowledge used to assess the assumptions or justify their validity	Methods (paragraphs 4)
8	**Sensitivity analyses and additional analyses**		Describe any sensitivity analyses or additional analyses performed (e.g. comparison of effect estimates from different approaches, independent replication, bias analytic techniques, validation of instruments, simulations)	Methods (paragraphs 4,5)
9	**Software and pre-registration**			
		a)	Name statistical software and package(s), including version and settings used	Methods (paragraph 6)
		b)	State whether the study protocol and details were pre-registered (as well as when and where)	N/A
	**RESULTS**			
10	**Descriptive data**			
		a)	Report the numbers of individuals at each stage of included studies and reasons for exclusion. Consider use of a flow-diagram	N/A

		b)	Report summary statistics for phenotypic exposure(s), outcome(s) and other relevant variables (e.g. means, SDs, proportions)	N/A
		c)	If the data sources include meta-analyses of previous studies, provide the assessments of heterogeneity across these studies	N/A
		d)	For *two-sample* Mendelian randomisation:<br>i. Provide justification of the similarity of the genetic variant-exposure associations between the exposure and outcome samples<br>ii. Provide information on the number of individuals who were in both samples for the exposure and for the outcome	Methods (paragraphs 2,3)
11	**Main results**			
		a)	Report the associations between genetic variant and exposure, and between genetic variant and outcome, preferably on an interpretable scale	N/A
		b)	Report MR estimates of the relationship between exposure and outcome, and the measures of uncertainty from the MR analysis, on an interpretable scale, such as odds ratio or relative risk per SD difference	Results (paragraphs 1-2), Figure 1
		c)	If relevant, consider translating estimates of relative risk into absolute risk for a meaningful time period	N/A
		d)	Consider plots to visualize results (e.g. forest plot, scatterplot of associations between genetic variants and outcome versus between genetic variants and exposure)	Figure 1
12	**Assessment of assumptions**			
		a)	Report the assessment of the validity of the assumptions	Results (paragraph 2)
		b)	Report any additional statistics (e.g., assessments of heterogeneity across genetic variants, such as I^2, Q statistic or E-value)	Results (paragraph 2, Figure 1, Tables S8-12)
13	**Sensitivity analyses and additional analyses**			
		a)	Report any sensitivity analyses to assess the robustness of the main results to violations of the assumptions	Results (paragraph 1,2) , Tables S8-12

		b)	Report results from other sensitivity analyses or additional analyses	Results (paragraphs 1, 2) , Tables S8-12
		c)	Report any assessment of direction of causal relationship (e.g., bidirectional MR)	Results (paragraph 2) , Table S12
		d)	When relevant, report and compare with estimates from non-MR analyses	N/A
		e)	Consider additional plots to visualize results (e.g., leave-one-out analyses)	N/A
	**DISCUSSION**			
14	**Key results**		Summarize key results with reference to study objectives	Discussion (paragraph 1)
15	**Limitations**		Discuss limitations of the study, taking into account the validity of the IV assumptions, other sources of potential bias, and imprecision. Discuss both direction and magnitude of any potential bias and any efforts to address them	Discussion (paragraph 5)
16	**Interpretation**			
		a)	Meaning: Give a cautious overall interpretation of results in the context of their limitations and in comparison with other studies	Discussion (paragraphs 2,3)
		b)	Mechanism: Discuss underlying biological mechanisms that could drive a potential causal relationship between the investigated exposure and the outcome, and whether the gene-environment equivalence assumption is reasonable. Use causal language carefully, clarifying that IV estimates may provide causal effects only under certain assumptions	Discussion (paragraphs 3)
		c)	Clinical relevance: Discuss whether the results have clinical or public policy relevance, and to what extent they inform effect sizes of possible interventions	Discussion (paragraph 4), Conclusion
17	**Generalizability**		Discuss the generalizability of the study results (a) to other populations, (b) across other exposure periods/timings, and (c) across other levels of exposure	Discussion (paragraph 4)
	**OTHER INFORMATION**			
18	**Funding**		Describe sources of funding and the role of funders in the present study and, if applicable, sources of funding for the databases and original study or studies on which the present study is based	N/A
19	**Data and data sharing**		Provide the data used to perform all analyses or report where and how the data can be accessed, and reference these sources in the article. Provide the statistical code needed to	Data availability section in Methods

		reproduce the results in the article, or report whether the code is publicly accessible and if so, where	
20	**Conflicts of Interest**	All authors should declare all potential conflicts of interest	Disclosure section
